# MetaMetaDB: A Database and Analytic System for Investigating Microbial Habitability

**DOI:** 10.1371/journal.pone.0087126

**Published:** 2014-01-27

**Authors:** Ching-chia Yang, Wataru Iwasaki

**Affiliations:** Atmosphere and Ocean Research Institute, the University of Tokyo, Kashiwa, Chiba, Japan; Keio University, Japan

## Abstract

MetaMetaDB (http://mmdb.aori.u-tokyo.ac.jp/) is a database and analytic system for investigating microbial habitability, i.e., how a prokaryotic group can inhabit different environments. The interaction between prokaryotes and the environment is a key issue in microbiology because distinct prokaryotic communities maintain distinct ecosystems. Because 16S ribosomal RNA (rRNA) sequences play pivotal roles in identifying prokaryotic species, a system that comprehensively links diverse environments to 16S rRNA sequences of the inhabitant prokaryotes is necessary for the systematic understanding of the microbial habitability. However, existing databases are biased to culturable prokaryotes and exhibit limitations in the comprehensiveness of the data because most prokaryotes are unculturable. Recently, metagenomic and 16S rRNA amplicon sequencing approaches have generated abundant 16S rRNA sequence data that encompass unculturable prokaryotes across diverse environments; however, these data are usually buried in large databases and are difficult to access. In this study, we developed MetaMetaDB (Meta-Metagenomic DataBase), which comprehensively and compactly covers 16S rRNA sequences retrieved from public datasets. Using MetaMetaDB, users can quickly generate hypotheses regarding the types of environments a prokaryotic group may be adapted to. We anticipate that MetaMetaDB will improve our understanding of the diversity and evolution of prokaryotes.

## Introduction

—*Everything is everywhere, but, the environment selects.* (Baas-Becking, 1934)

Prokaryotes are the most numerous and diverse organisms on the planet, and distinct prokaryotic communities maintain and characterize distinct ecosystems [1]. Therefore, investigating how prokaryotes adapt to each environment (a primary question in the field of microbial ecology) is pivotal in delineating our biosphere and its dynamics. To answer the question of *microbial habitability*, it is necessary to identify comprehensively the types of environments in which any group of prokaryotes exists. With this aim, a general method to identify prokaryotic species in natural environments is to obtain the 16S ribosomal RNA (rRNA) sequences of those species, which are essential and highly conserved in all prokaryotes and are ideal for the taxonomical assignment of prokaryotes. However, individual efforts to culture prokaryotic strains or sample 16S rRNA sequences exhibit limitations in uncovering the diversity of the environments that any group of prokaryotes can live in. In other words, to understand systematically microbial habitability, it is necessary to collect and analyze 16S rRNA data from as many environments as possible.

There are several existing 16S rRNA data collections, such as Greengenes [2], the Ribosomal Database Project [3], and SILVA [4], which contain thousands of 16S rRNA sequences from a wide range of prokaryotes. These 16S rRNA sequences are well annotated in taxonomy; however, the information regarding the environments these prokaryotes are found in is lacking in these databases, and the prokaryote-environment interactions cannot be readily analyzed. Moreover, although the 16S rRNA sequences of uncultured prokaryotes are also included in the databases, the collections are biased to culturable species. This issue cannot be ignored because it is estimated that over 99% of prokaryotes are unculturable [5]–[7].

Because many prokaryotes cannot be cultured in the laboratory, whole-genome shotgun metagenomic and 16S rRNA amplicon sequencing studies that acquire the genetic information of entire microbial communities directly from environmental samples have become popular [8], [9]. In recent years, the rapid advancements in deep sequencing technologies in particular have dramatically decreased the cost of sequencing, and sequencing speed has increased by several orders of magnitude [10], [11]. Thanks to these new technologies, we now have billions of prokaryotic genetic sequences from diverse environments; however, these data are buried in large databases and are difficult to access. Although a number of analytic systems incorporate metagenomic data, such as MEGAN [12], MG-RAST [13], and IMG/M [14], these systems do not provide a compact dataset of 16S rRNA sequences with environmental information.

In this study, we developed MetaMetaDB, a comprehensive (“meta-”) and compact database that collects 16S rRNA sequences from a large number of datasets. To ease the difficulties of analyzing a large amount of data, we strategically extracted the genetic information and made a comprehensive but compact collection of 16S rRNA sequences that are associated with diverse environments. By submitting the 16S rRNA sequences of certain prokaryotes to MetaMetaDB, users can easily investigate the microbial habitability for analyzing the ecology and evolution of prokaryotes.

## Materials and Methods

### Data source

To create a comprehensive 16S rRNA dataset that covers both whole-genome shotgun metagenomic and 16S rRNA amplicon sequencing projects, we first downloaded all the sequence data whose study type was *metagenomics* from the DDBJ Sequence Read Archive (DRA) [15] (Table S1) on January 11, 2013. The principal sequencing platforms for these datasets were Illumina and 454; however, the data derived from the Illumina platforms were extremely biased to a specific environment (e.g., the human gut) and contained sequences that were too short for taxonomic identification. Therefore, we extracted only data derived from the 454 platform, which has been used by a wide range of projects covering diverse environments. The resulting 4,606 files, containing 181,416,932 sequences, were used as raw data. Next, every sequence was linked to an *environmental category*, which was retrieved from the *scientific name* metadata of the associated sequencing project entries in DRA. We adopted this metadata because these data were based on the NCBI Taxonomy hierarchy [16], which contains terms of environmental classifications and species taxonomic classifications and could therefore be used as a controlled vocabulary in the database. The list of the environmental categories used in our database is shown in Table 1.

**Table 1:** The number of representative 16S rRNA sequences in each environmental category.

Environment	Number of files	16S rRNA sequences	representative 16S rRNA sequences	Average (bp)	Min (bp)	Max(bp)
ant fungus garden	127	68,118	11,556	393.07	200	576
ant	9	10,915	3,436	377.50	200	583
beach sand	4	2,038	1,234	357.52	200	538
beetle	19	11,895	1,708	397.53	200	561
biofilm	29	24,317	3,723	449.99	204	806
bioreactor	39	181,862	33,591	436.26	200	620
bioreactor sludge	226	286,831	50,666	242.91	200	541
bovine gut	16	319,384	59,564	362.20	200	655
chicken gut	5	3,579	1,496	265.18	200	406
compost	24	14,914	2,195	381.07	200	599
coral	14	589	192	289.62	200	467
fish	26	87,742	49,706	420.24	200	861
food fermentation	1	90,024	51,908	380.78	200	673
food	15	79,971	37,250	399.89	200	915
freshwater	34	102,460	35,880	351.29	200	797
freshwater sediment	2	9,368	7,128	404.95	200	803
groundwater	1	4,524	1,296	365.04	200	550
gut	90	299,036	43,670	288.22	200	621
honeybee	2	34,323	23,479	377.10	200	820
hot springs	13	10,312	2,646	375.05	200	572
human gut	247	216,566	64,787	252.06	200	592
human	1,272	311,651	53,834	365.09	200	842
human oral	257	242,633	11,574	287.97	200	547
human skin	173	55,863	22,114	362.43	200	866
hydrocarbon	506	413,879	46,431	413.31	200	704
hydrothermal vent	1	739	235	251.52	200	296
hypersaline lake	5	6,957	2,474	399.30	200	548
ice	3	456	421	249.59	200	312
marine	823	79,871	27,447	294.65	200	582
marine sediment	80	21,155	9,393	342.97	200	680
microbial mat	4	4,561	1,930	247.02	201	313
mine drainage	4	1,217	674	357.20	200	553
mosquito	24	46,888	4,324	375.05	200	563
mouse gut	20	37,536	11,021	247.95	200	512
oil production facility	17	4,893	1,691	232.90	200	523
phyllosphere	35	2,108	1,199	263.95	201	583
rhizosphere	13	67,371	17,514	374.79	200	565
root	2	7,260	1,590	377.01	200	512
saltern	15	2,309	651	393.71	200	570
sediment	89	137,175	37,739	387.37	200	772
soil	257	1,775,170	490,776	341.19	200	806
stromatolite	7	17,441	2,633	246.28	202	266
termite gut*	1	-	-	-	-	-
termite	8	12636	1557	388.31	200	732
wasp	7	8602	616	369.08	200	565
wastewater	40	20373	6264	373.78	200	526
**Total**	**4,606**	**5,137,512**	**1,241,213**	**346.82**	**200**	**915**

* All the reads from the termite gut were shorter than 200 bp, and the average length was not calculated.

“Average” represents the average length, whereas “Min” and “Max” represent the lengths of the shortest and the longest sequences.

### Data processing

**Figure 1:**
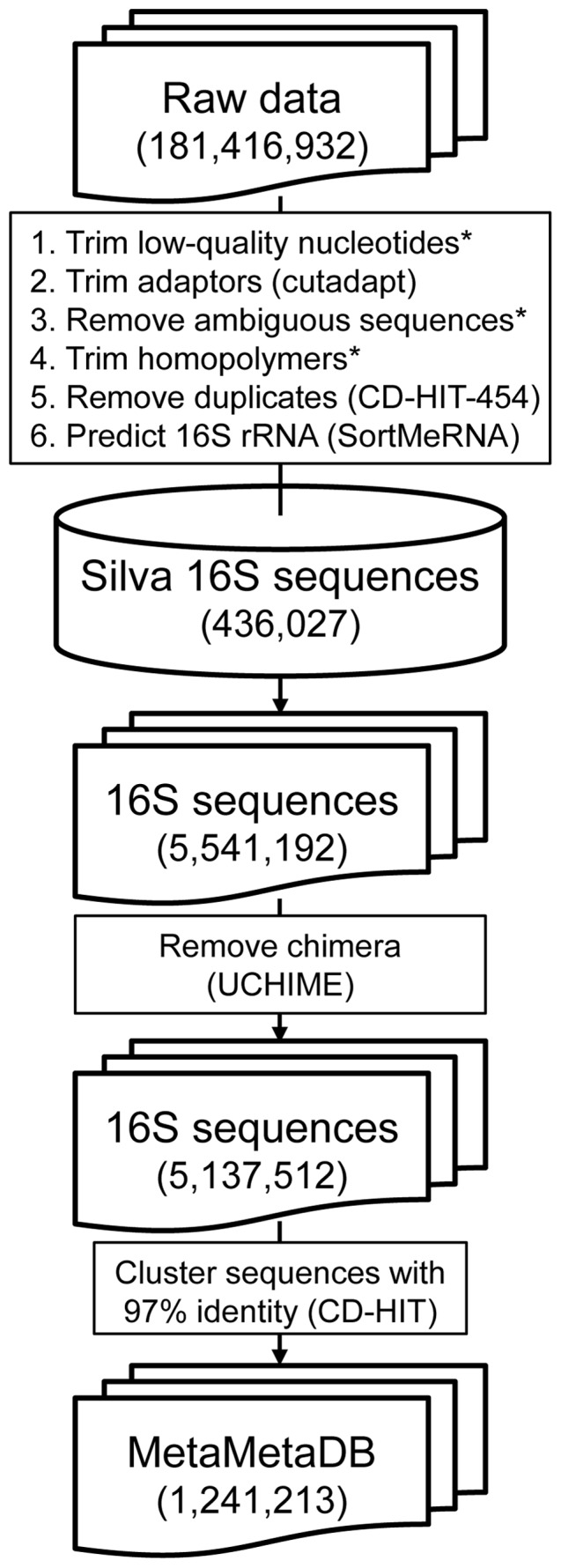
Flowchart for generating the MetaMetaDB content. A large amount of data was effectively condensed into a small number of representative 16S rRNA sequences. The asterisks indicate the processes done by NGS QC Toolkit. The numbers in the parentheses represent the numbers of sequences. The tool used in each step is described in the parentheses of that step.

A fundamental challenge of data processing lies in compressing a large number of metagenomic sequences into a small collection without losing much information. Although simply running a sequence similarity search against the above raw data might provide information regarding the environmental categories where a given prokaryotic group can inhabit, the data should be processed in advance to serve as a ready-to-use system for microbiologists. First, the raw data contained non-16S rRNA sequences derived not only from the whole-genome shotgun metagenomes but also from the 16S rRNA amplicon sequencing data. Second, the raw data contained many redundant sequences of identical or highly similar prokaryotes because of the quantitative nature of the metagenomic sequencing, repetitive samplings in similar environments, and artificial sequence duplications generated during the sample preparation steps. All these factors made the data size too large for the rapid return of the search results, and the search results occupied by similar sequences from the same environmental category masked the true habitat diversity of the prokaryotic group of interest. It should be also noted that, although the numbers of 16S rRNA sequences would reflect volumes of prokaryotic groups in a single metagenomic study, these numbers cannot be directly used in meta-analysis because volumes of studies in each environmental category do not reflect volumes of prokaryotic groups in each environmental category on the planet. Therefore, we performed several steps, which are summarized in Figure 1, to generate a compact 16S rRNA sequence collection for inferring microbial habitability.

First, low-quality nucleotides with Phred-equivalent quality scores under 20 were trimmed using TrimmingReads.pl of NGS QC Toolkit [17], and then Cutadapt (version 1.1) [18] was performed to remove the adaptor sequences (Steps 1 and 2 in Figure 1). We removed sequences with ambiguous nucleotides by AmbiguityFiltering.pl and trimmed homopolymers that are 5 bp or longer by HomopolymerTrimming.pl of NGS QC Toolkit, respectively (Steps 3 and 4 in Figure 1). Sequences without Phred scores were removed from the dataset. We used CD-HIT-454 [19] to remove possible artificial duplicates that were generated in sequencing processes, especially those from 16S rRNA amplicon sequencing [20]. The identity for using CD-HIT-454 was 99% for tolerating sequencing errors. In each of the trimming steps, we removed sequences that were shorter than 200 bp. Generally, sequence similarity searches against these short sequences result in low bit scores; therefore, these sequences rarely appear in search results and have little effect. However, these rarely matched sequences could cause a bias in the normalization process that would lead to a long computation time for the search. In addition, a 16S rRNA sequence includes variable and conserved regions, and only the variable regions are informative [21]; therefore, a 16S rRNA sequence fragment containing only conserved regions would be assigned to many species and lead to an incorrect inference. Because conserved regions are usually shorter than 200 bp [22], the sequences containing only conserved regions would be excluded from the dataset in this step. Second, we predicted possible 16S rRNA sequences using SortMeRNA (version 1.8) [23] with the non-redundant reference database from SILVA (release 115) [4], which includes 418,497 bacterial and 17,530 archaeal 16S rRNA sequences. In total, 5,541,192 16S rRNA sequences were obtained. Third, we remove the chimera sequences by UCHIME [24] with the suggested reference database because the chimera sequences account for 5 to 45% of 16S amplicon sequences [20], [25], which may cause erroneous results in sequence comparison. After this step, we acquired 5,137,512 16S rRNA sequences without possible experimental bias. Fourth, to remove the redundant sequences, we applied CD-HIT [26] to cluster our 16S rRNA sequences with 97% identity within each environmental category as 97% identity is the accepted boundary for prokaryotic species [27]. Because the raw data contained sequences of different 16S rRNA regions derived from the whole-genome shotgun metagenomes and 16S rRNA amplicon sequencing using different PCR primers, the 97% threshold can still keep multiple sequences of the same species in each environmental category. Finally, we obtained a compact “meta-metagenomic” dataset with 1,241,213 rRNA sequences that were each marked by one of the 45 environmental categories (Table 1). Taxonomic information of the 16S rRNA sequences was obtained by Ribosomal Database Project (RDP) Classifier (version 2.5) [28] with the cutoff of 50%, the output format of “filterbyconf”, and the default training datasets.

### Calculation of the Microbial Habitability Index

We designed the usage of MetaMetaDB to be intuitive; users simply submit a 16S rRNA sequence from a prokaryote of interest to obtain the results. MetaMetaDB receives the query sequence, runs a BLASTN (BLAST+ version 2.2.26) [29] search against the meta-metagenomic 16S rRNA dataset with the e-value of 1e-10, and generates a list of hits. Because BLAST does not provide an option to sort outputs by identity, MetaMetaDB receives at most 10,000 significant hits (in terms of e-value) from the list of BLASTN hits and sorts the hits by the ratio of the number of aligned nucleotides to the summation of the length of the hit sequence and gap(s) that appear in the hit sequence. Thus, the query sequence should be longer than the hit sequences, and we recommend more than 500 bp for the present version of the database. Because the sequences in MetaMetaDB are marked by environmental categories, the significant hits suggest environmental categories in which the taxonomically related prokaryotes inhabit. Accordingly, MetaMetaDB calculates the Microbial Habitability Indices (MHIs) as a set of (normalized) ratios of environmental categories among the significant hits. It is intrinsically difficult to set one threshold on the identity because the identity depends on the evolutionary distance between the prokaryotic groups that the users want to take into account. Therefore, MetaMetaDB chooses the hits exhibiting above 97%, 95%, 90%, 85%, and 80% identity from the search result list, which correspond approximately to the taxonomic levels of species, genus, family, order, and class [1], respectively, and calculates the ratios of the environmental categories in each identity level. Because the numbers of 16S rRNA sequences in the entire meta-metagenomic dataset are different between the environmental categories (Table 1), the categories with more sequences are expected to appear more frequently in those lists by chance. Therefore, we adopted a logarithmic weighting factor that resembles the inverse document frequency. The inverse document frequency is widely used in the text mining fields, where terms that frequently occur in document collections are assigned less weight. Mathematically, the MHI for each environmental category *e* with an identity threshold *c* is defined by
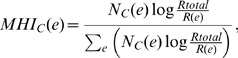



where *N*_c_(*e*) is the number of hits that are marked by *e* and above identity *c*, *R_total_* is the total number of sequences in the database (1,241,213 in the current version of the database), and *R*(*e*) is the total number of sequences marked by *e* in the database. Larger MHI(*e*) reflects larger possibility to be found in the environment *e*; however, note that it is not the exact probability that can be directly compared with other MHIs by its value.

Users can also submit multiple intact 16S rRNA sequences to MetaMetaDB. In this case, users can analyze these sequences either individually or collectively. In the former case, MHIs are calculated for each query sequence. In the latter case, the number of hits that are marked by *e* and above identity *c* from each query sequence is summed up to be *N*_c_(*e*) used in the fore-mentioned MHI calculation. MHIs are averaged across all query sequences to infer the habitability as a group of prokaryotes or summarize results for multiple 16S rRNA sequences from the same species.

## Results

### Usage Example

**Figure 2:**
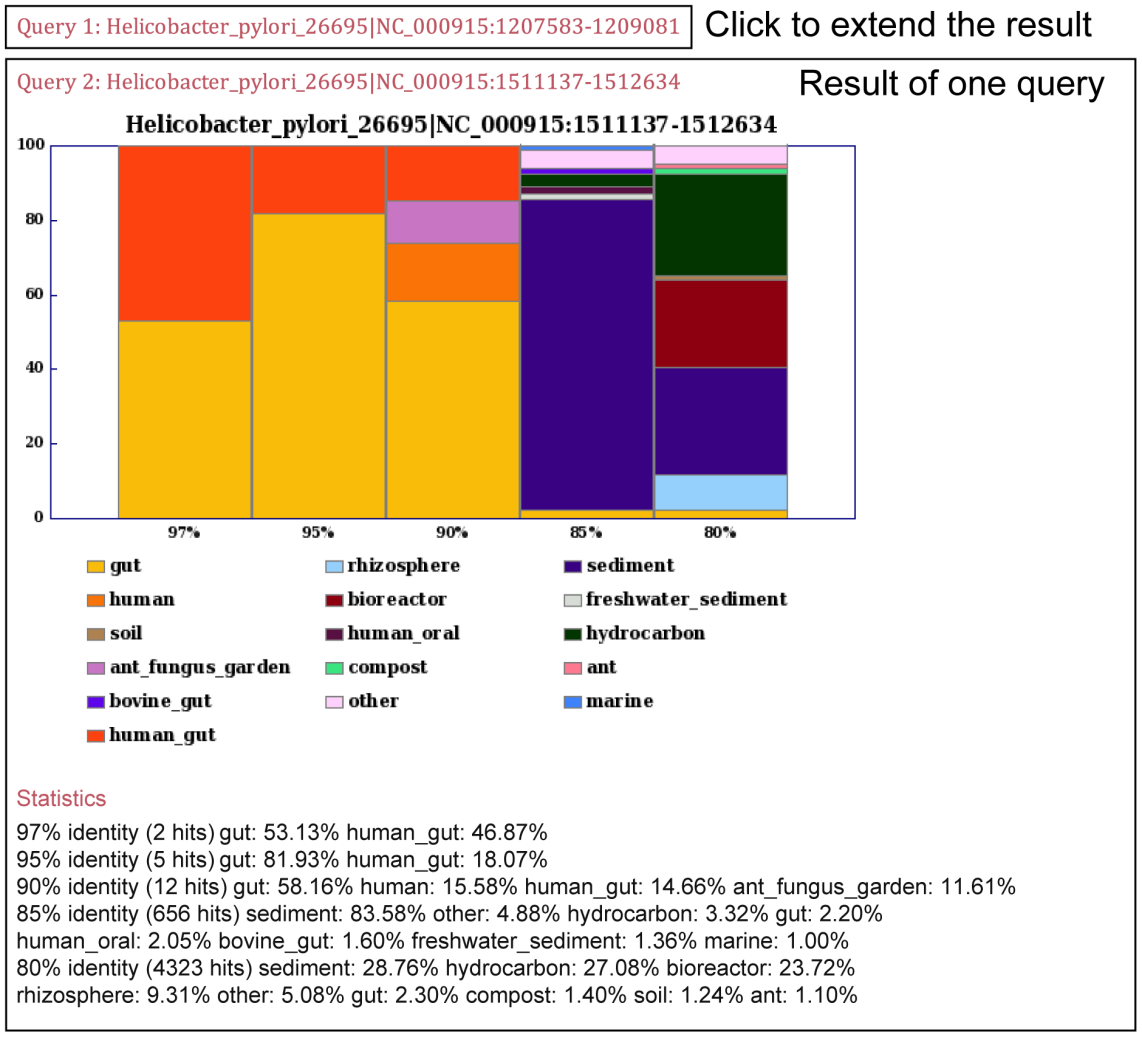
Screen image of a sample results page. MetaMetaDB produces one figure and one statistical description for each query sequence and provides a link to download all of the figures and text files. The y-axis represents the MHIs, and the columns show the MHIs calculated by the hits above the different identities. The MHIs and the number of hit(s) used for the calculation are listed under the figure.

Users can submit sequences to MetaMetaDB using a text area in the homepage. After the BLASTN search and the MHI calculations are performed, a webpage presenting the results appears as shown in Figure 2 (the sample query was the *Helicobacter pylori* 26695 16S rRNA sequence obtained from its genome [30]). A typical waiting time is approximately 15 seconds if one 1,500-bp 16S rRNA sequence is queried, thus the system can be readily utilized for quick analyses. In the bar-plot figure, the *y*-axis shows the MHI of each environmental category listed at the bottom of the figure. The columns labeled *97%*, *95%*, *90%*, *85%*, and *80%* represent the thresholds of identities used in the calculation. The summation is 100% for all columns by definition. The environments with MHIs that are less than 1% in each column are summarized and are labeled as *other*. If there is no hit above any identity threshold, then the corresponding column does not appear. In addition to the bar-plot figure, users can also obtain numeric data below the figure or by clicking *Statistics* (for example, the MHI based on the hits with greater than 95% identity in gut-associated environments were 81.93%). In the case of *H. pylori*, a human pathogen that causes gastric inflammation and peptic ulcer disease [30], based on a comprehensive metagenomic dataset, the MHI bar-plots confidently suggest that gut-associated environments are where this prokaryotic group is uniquely found. These data are consistent with the data from the pre-deep-sequencing era demonstrating that *H. pylori* rarely exists outside human gastrointestinal tracts [31]. At the family level (90%), this figure also shows different environmental category of ant fungus gardens, suggesting possible habitat of related groups of *H. pylori*. An example of the collective analysis of multiple query sequences is given in Figure S1 in which two 16S rRNA sequences of *Helicobacter pylori* 26695 were queried. The interpretation of the output was the same as the outputs generated for each query sequence.

For users who wish to analyze data on personal servers, we allow the users to download the meta-metagenomic data in the conventional FASTA format by providing a compressed FASTA file that contains all of the reads marked by the environmental categories. The reads for each environmental category E are labeled as E.1, E.2, E.3, etc. In addition, for users who are interested in further taxonomic information of the hit sequences, we provide a tab-delimited text file of taxonomic descriptions for all the sequences for downloading. We also provide pre-calculated MHIs of 16S rRNA sequences from the All-Species Living Tree project [32] (release 111) of SILVA for users link microbial habitability to the species that are well-characterized in taxonomy.

### Evaluation of MetaMetaDB

We examined four well-studied species to evaluate the microbial habitability inferred by MetaMetaDB. We chose *Methanothermobacter thermautotrophicus* str. Delta H [33], *Bacillus subtilis* 168 [34], *Escherichia coli* str. K-12 substr. MG1655 [35], and *Vibrio cholerae* El Tor N16961 [36], which are well-known archaeal and bacterial species with genomic sequences that were determined in the early stages of the genomic era. All the full-length 16S rRNA genes encoded in each genome were submitted to MetaMetaDB to collectively calculate the MHIs. The MHIs and the known habitats of the species are summarized in Table 2 and Figure S2. The MHIs and the known habitats were largely consistent in these species. *M*. *thermautotrophicus* was isolated from sewage sludge [37], which is consistent with the high MHI of the wastewater and compost category (Figure S2A). Interestingly, the human skin category also appears in the bar plots, which may imply a relationship with human. *B*. *subtilis* can be found in the soil, water sources and in association with plants [34], and *E. coli* colonizes the lower gut of animals and can survive in the natural environment, which allows for widespread dissemination to new hosts [35]. The MHIs of *B*. *subtilis* (Figure S2B) and *E. coli* (Figure S2C) suggest that these species may occupy wide ranges of natural and animal-associated environments. *V. cholerae* has been known to inhabit in aqua environments [36], where MHIs precisely show marine and freshwater environments (Figure S2D).

**Table 2:** Habitats and MHIs inferred by MetaMetaDB.

Domain	Species	Habitat*	Number of 16S rRNA	MHI**
Archaea	*Methanothermobacter thermautotrophicus*	Wastewater, Sludge	2	(97%, 14 hits) wastewater: 59.12%; compost: 17.71%; rhizosphere: 11.91%; human skin: 11.26%
Bacteria	*Bacillus subtilis*	Soil	10	(97%, 164 hits) hydrocarbon: 23.68%; wastewater: 19.89%; root: 10.44%; compost: 8.94%; ant fungus garden: 7.33%; rhizosphere: 6.68%; food: 5.49%; sediment: 5.47%; fish: 5.04%; gut: 4.72%; soil: 2.33%
Bacteria	*Escherichia coli*	Human intestinal microflora, Host	7	(97%, 1,032 hits) hydrothermal vent: 33.66%; termite: 14.31%; freshwater: 8.16%; other: 7.52%; ant fungus garden: 5.17%; marine: 4.63%; microbial mat: 4.50%; gut: 4.42%; beetle: 4.12%; human gut: 3.48%; phyllosphere: 3.10%; ant: 2.31%; wastewater: 1.79%; hydrocarbon: 1.47%; sediment: 1.37%
Bacteria	*Vibrio cholerae*	Wastewater, Intestinal microflora, Host, Fresh water	8	(97%, 36 hits) freshwater: 88.15%; marine: 11.85%
Bacteria	*Helicobacter pylori* 26695	Human stomach, Host	2	(97%, 3 hits) human gut: 63.82%; gut: 36.18%

* The habitats were retrieved from the Genomes OnLine Database (GOLD) [40].

** Only MHIs that were calculated by hits above highest identity threshold are listed.

These four examples and the *H. pylori* sample demonstrated that MetaMetaDB not only reflects the habitats of prokaryotes but also provides further directions for the investigation of microbial ecology and evolution. For example, future studies could include the isolation of similar prokaryotic strains from different environments to reveal the genetic bases for new habitat adaptation. Additionally, we can extend our understanding of microbial ecology to a wider scale as we can input multiple query sequences of a higher taxonomic group (genus, family, etc.) or a given microbial community.

## Discussion

Metagenomic studies have become routine in the field of microbiology, and novel resources to extract efficiently as much knowledge as possible from the large datasets would provide new ways to deepen our understanding of the microbial world. To date, metagenomic data have been analyzed from the perspective of the components of each microbial community; however, MetaMetaDB provides a reverse perspective of the environments in which each prokaryotic group exists, opening the door to the investigation of “meta-metagenomics”.

MetaMetaDB uses 45 categories that describe the sampling environments, mainly because there was no alternative consistent terminology that is linked to all sequence data in DRA. We assumed that this number of categories would be effective for evaluating the overall tendencies of prokaryotic habitat preferences; however, this number may be insufficient for analyzing microbial ecologies on finer scales. For example, the composition of the microbes in the Sargasso Sea is different from that in the Arctic Ocean [38] but these environments are clustered in the *marine* category in MetaMetaDB. Another possible issue is that one sample could be assigned to several scientific names in DRA; for example, a sample classified as *marine sediment* can also be assigned to *marine* or *sediment*, depending on the data submitter. In the future, we plan to adopt a determinate vocabulary for environments to connect precisely and unambiguously the microbial communities and the environmental features that shaped these communities. For example, MIGS/MIMS [39], EnvO (http://environmentontology.org/home), and Metagenome/Microbes Environment Ontology (MEO, http://mdb.bio.titech.ac.jp/meo) are candidate vocabulary resources that generate clear definitions of environmental terms and the structured relationships between these terms.

Future changes could also be applied in the data-processing steps. We used 16S rRNA sequences longer than 200 bp in the current database to increase the coverage as much as possible; however, a longer threshold may be preferable to identify prokaryotes with more accuracy. In addition, limiting regions of the 16S rRNA sequences could effectively avoid the effects from different sequence conservation patterns across different regions. In this case, a substantial obstacle is that limiting the regions can make the data coverage much smaller because there are many universal PCR primer sets for 16S rRNA amplicon sequencing. Additionally, 16S rRNA sequences from the same sample can be assembled before incorporation into the database. In this study, we skipped the assembly process because the aim of MetaMetaDB is not to precisely identify closely related species, which usually requires long 16S rRNA sequences. As technical advancements provide longer and more abundant metagenomic sequences, we plan to update the database every six months for better and more precise usage.

Several future perspectives are envisioned based on the present study. First, the evolution of microbial habitability is of great interest because prokaryotes are known to change habitats frequently. Second, the relationship between the genome and microbial habitability is also to be investigated because this relationship will provide answers to why specific groups of prokaryotes can adapt to more diverse environments than other groups of prokaryotes. Third, increased knowledge of microbial habitability could contribute to the applications of agricultural, industrial, medical, and environmental sciences. We anticipate increased efforts in the development of analytic tools for this “meta-metagenomics” field.

## Supporting Information

Figure S1 **A screen shot of analysis process for multiple sequences.**
(PDF)

Figure S2 **MHIs of four selected examples.**
(PDF)

Table S1 **Accession numbers of sequence run files used in MetaMetaDB.**
(PDF)
