# Supplementary material for: MetaMetaDB: A Database and Analytic System for Investigating Microbial Habitability
Source: PLoS One. 2014 Jan 27;9(1):e87126. doi: 10.1371/journal.pone.0087126 (PMC3903645; doi:10.1371/journal.pone.0087126)

Figure S1. A screen shot of analysis process for multiple sequences. MetaMetaDB produces one figure and one statistical description for all the query sequences and provides a link to download the figure, the text file, and the BLAST result. Two example sequences were downloaded from *Helicobacter pylori* 26695 genome in NCBI.

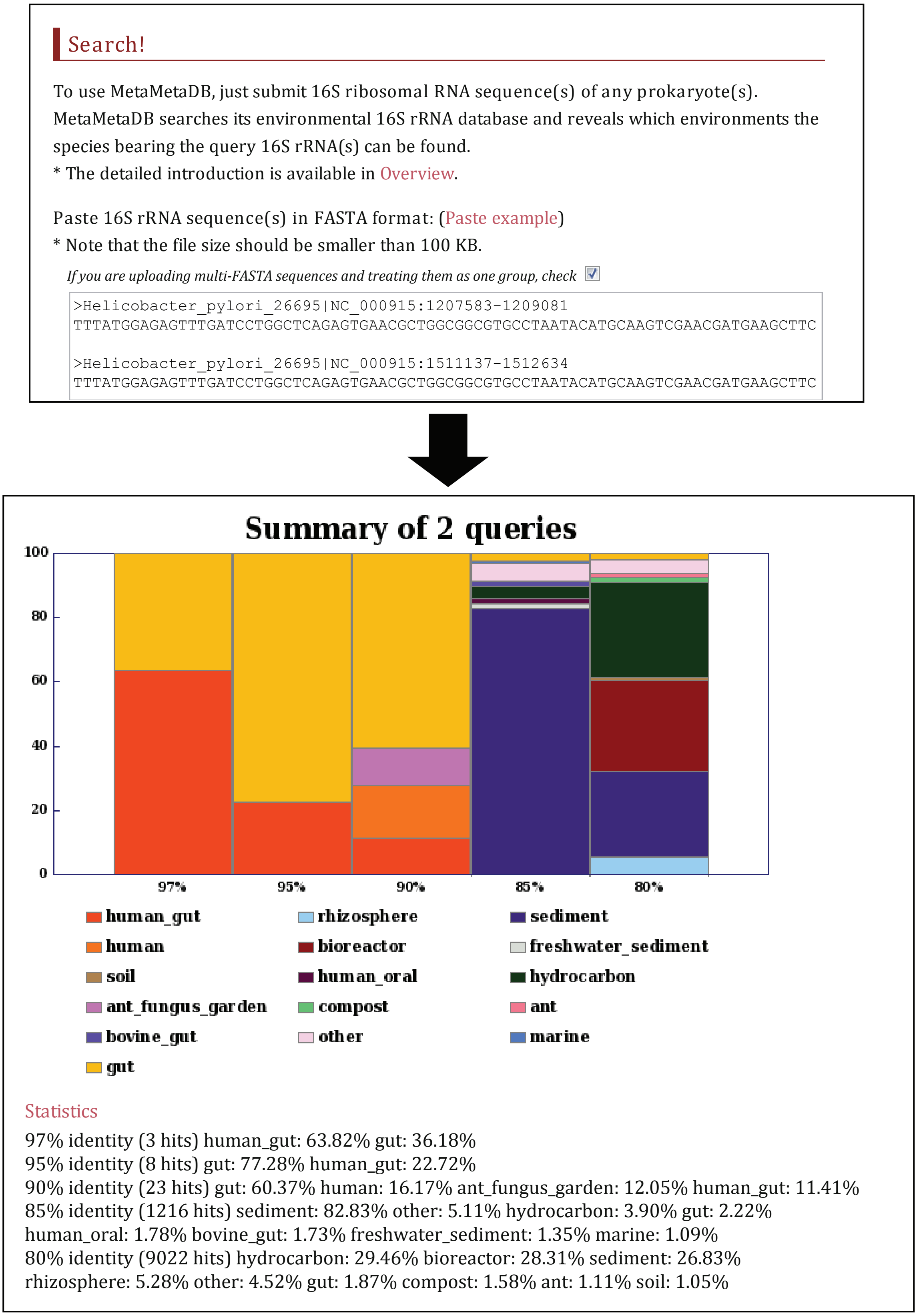

Supplement: Figure S1 — A screen shot of analysis process for multiple sequences. (PDF) [file pone.0087126.s001.pdf]
