# Supplementary material for: MetaMetaDB: A Database and Analytic System for Investigating Microbial Habitability
Source: PLoS One. 2014 Jan 27;9(1):e87126. doi: 10.1371/journal.pone.0087126 (PMC3903645; doi:10.1371/journal.pone.0087126)
Supplement: Figure S2 — MHIs of four selected examples. (PDF) [file pone.0087126.s002.pdf]

Figure S2. MHIs of four selected examples. (A) *Methanothermobacter thermautotrophicus* (B) *Bacillus subtilis* (C) *Escherichia coli* (D) *Vibrio cholerae*.

(A)

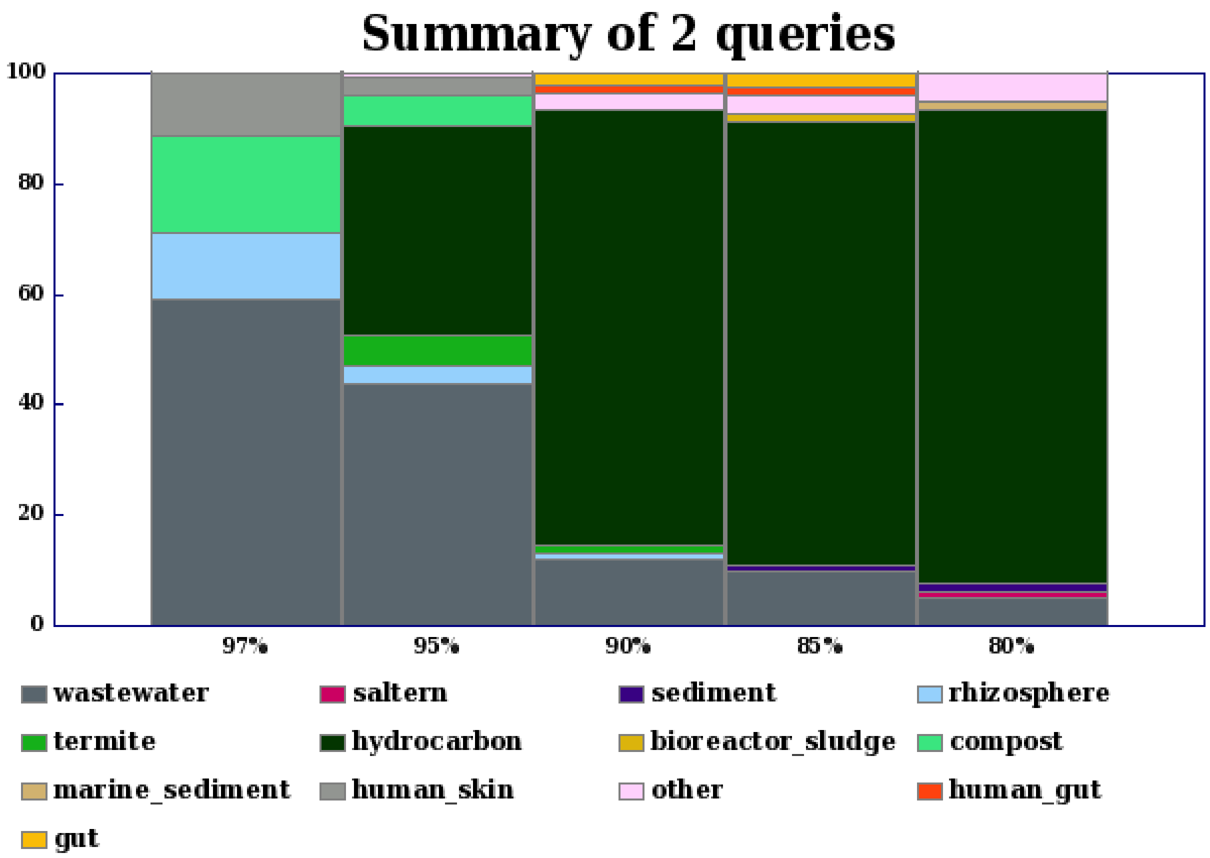

97% identity (14 hits) wastewater: 59.12% compost: 17.71% rhizosphere: 11.91% human_skin: 11.26%
95% identity (58 hits) wastewater: 43.66% hydrocarbon: 37.98% termite: 5.52% compost: 5.23% rhizosphere: 3.52% human_skin: 3.33% other: 0.77%
90% identity (511 hits) hydrocarbon: 78.88% wastewater: 11.87% other: 2.98% gut: 2.25% termite: 1.50% human_gut: 1.32% rhizosphere: 1.19%
85% identity (1272 hits) hydrocarbon: 80.22% wastewater: 9.91% other: 3.20% gut: 2.68% human_gut: 1.48% bioreactor_sludge: 1.46% sediment: 1.04%
80% identity (4757 hits) hydrocarbon: 85.75% other: 5.25% wastewater: 5.01% sediment: 1.38% saltern: 1.35% marine_sediment: 1.26%

(B)

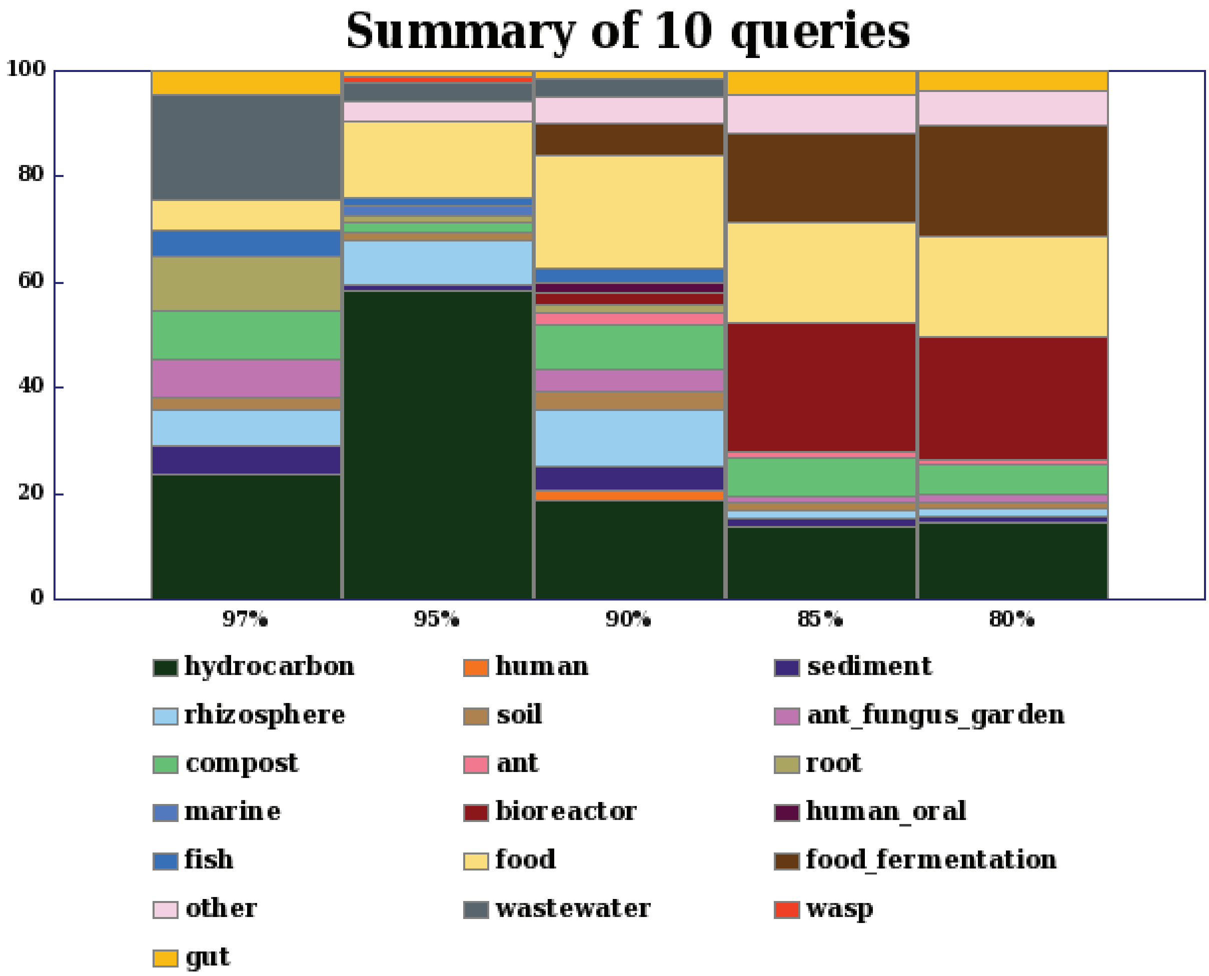

97% identity (164 hits) hydrocarbon: 23.68% wastewater: 19.89% root: 10.44% compost: 8.94% ant_fungus_garden: 7.33% rhizosphere: 6.68% food: 5.49% sediment: 5.47% fish: 5.04% gut: 4.72% soil: 2.33%
95% identity (1830 hits) hydrocarbon: 58.42% food: 14.49% rhizosphere: 8.26% other: 4.00% wastewater: 3.42% compost: 2.05% marine: 1.85% soil: 1.66% fish: 1.35% wasp: 1.23% sediment: 1.13% root: 1.08% gut: 1.08%
90% identity (11423 hits) food: 21.47% hydrocarbon: 18.71% rhizosphere: 10.39% compost: 8.51% food_fermentation: 6.20% other: 4.91% sediment: 4.72% ant_fungus_garden: 4.20% soil: 3.45% wastewater: 3.42% fish: 2.47% ant: 2.20% bioreactor: 2.19% human_oral: 2.08% human: 1.92% root: 1.68% gut: 1.48%
85% identity (74400 hits) bioreactor: 24.41% food: 18.87% food_fermentation: 16.94% hydrocarbon: 13.80% compost: 7.19% other: 7.14% gut: 4.62% rhizosphere: 1.73% soil: 1.39% ant_fungus_garden: 1.34% sediment: 1.30% ant: 1.25%
80% identity (97458 hits) bioreactor: 23.19% food_fermentation: 21.16% food: 19.01% hydrocarbon: 14.69% other: 6.55% compost: 5.73% gut: 3.66% soil: 1.36% rhizosphere: 1.35% ant_fungus_garden: 1.18% sediment: 1.09% ant: 1.03%

(C)

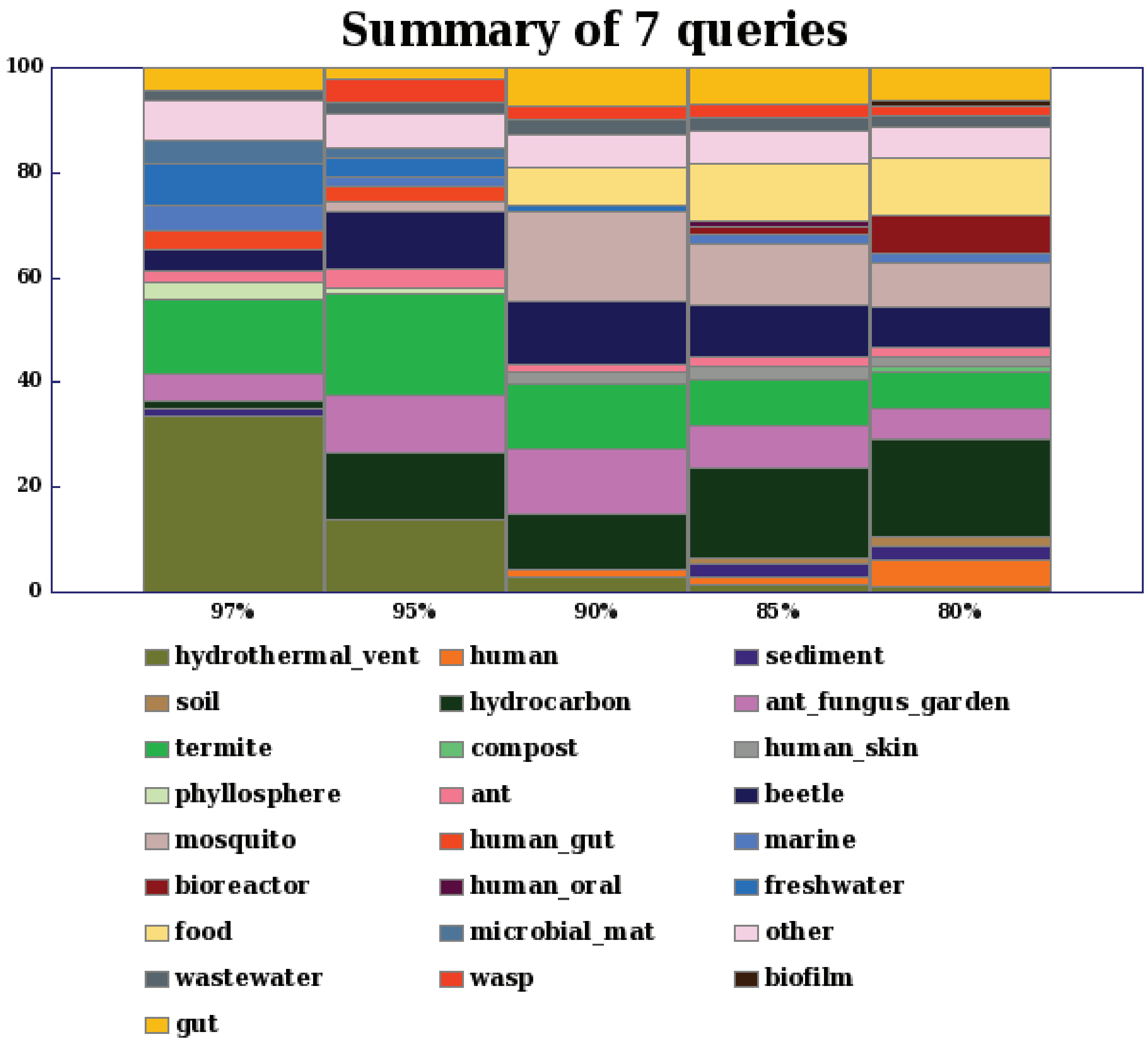

97% identity (1032 hits) hydrothermal_vent: 33.66% termite: 14.31% freshwater: 8.16% other: 7.52% ant_fungus_garden: 5.17% marine: 4.63% microbial_mat: 4.50% gut: 4.42% beetle: 4.12% human_gut: 3.48% phyllosphere: 3.10% ant: 2.31% wastewater: 1.79% hydrocarbon: 1.47% sediment: 1.37%
95% identity (2846 hits) termite: 19.18% hydrothermal_vent: 13.72% hydrocarbon: 12.93% beetle: 11.01% ant_fungus_garden: 10.98% other: 6.79% wasp: 4.45% freshwater: 3.72% ant: 3.32% human_gut: 2.87% gut: 2.15% wastewater: 2.05% mosquito: 1.84% marine: 1.83% microbial_mat: 1.78% phyllosphere: 1.37%
90% identity (14502 hits) mosquito: 16.94% termite: 12.48% ant_fungus_garden: 12.43% beetle: 12.37% hydrocarbon: 10.57% food: 7.44% gut: 7.29% other: 6.26% hydrothermal_vent: 2.98% wastewater: 2.89% wasp: 2.54% human_skin: 2.19% human: 1.41% ant: 1.19% freshwater: 1.01%
85% identity (29199 hits) hydrocarbon: 17.13% mosquito: 11.59% food: 11.09% beetle: 9.84% termite: 9.03% ant_fungus_garden: 7.84% gut: 6.93% other: 6.30% wastewater: 2.54% sediment: 2.53% wasp: 2.49% human_skin: 2.33% ant: 2.02% marine: 1.65% hydrothermal_vent: 1.64% bioreactor: 1.51% human: 1.38% soil: 1.13% human_oral: 1.01%
80% identity (46069 hits) hydrocarbon: 18.76% food: 11.17% mosquito: 8.47% beetle: 7.64% bioreactor: 7.21% termite: 6.97% gut: 6.04% ant_fungus_garden: 5.79% other: 5.66% human: 4.92% sediment: 2.80% wastewater: 2.14% human_skin: 1.90% wasp: 1.88% marine: 1.84% ant: 1.71% soil: 1.69% bio⌧ilm: 1.29% hydrothermal_vent: 1.12% compost: 1.01%

(D)

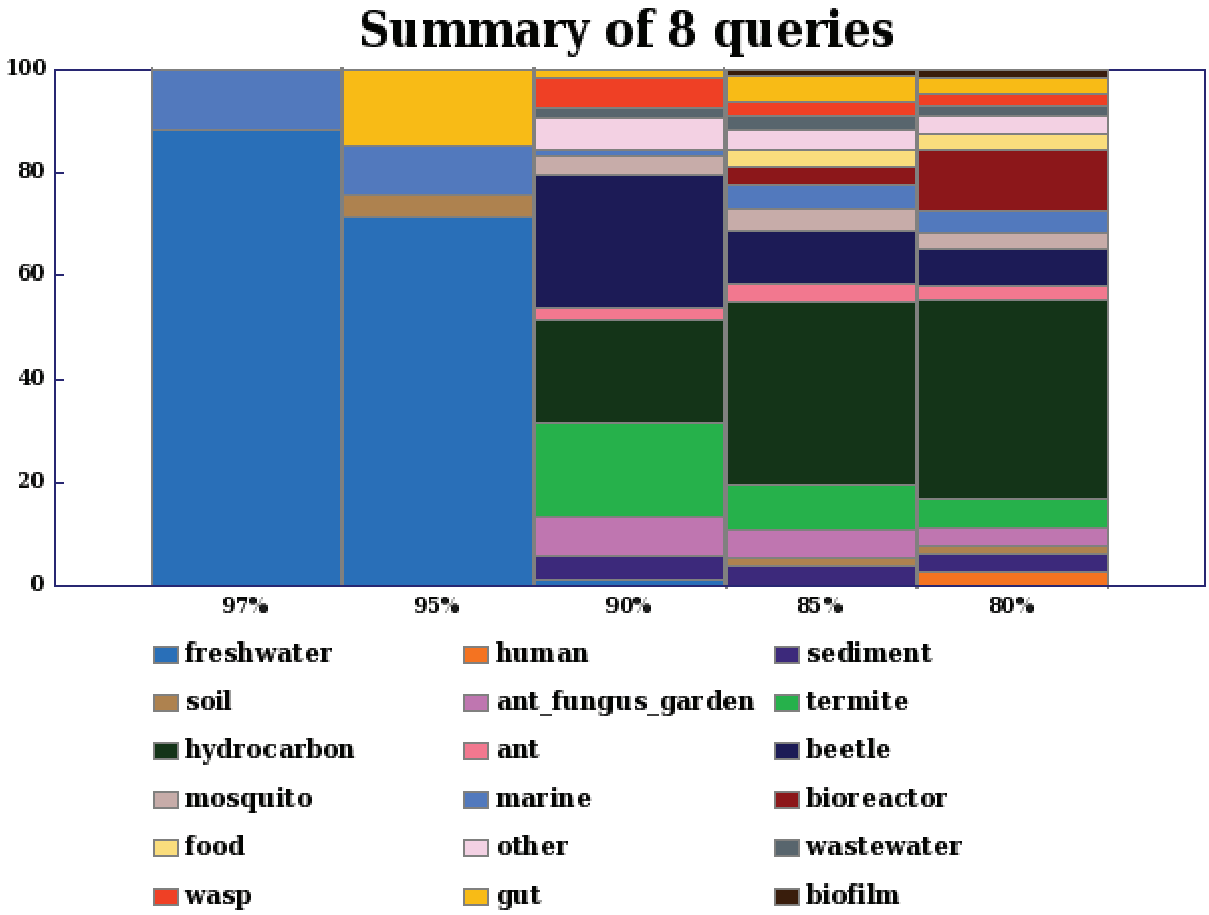

97% identity (36 hits) freshwater: 88.15% marine: 11.85%
95% identity (50 hits) freshwater: 71.51% gut: 14.78% marine: 9.61% soil: 4.10%
90% identity (3748 hits) beetle: 25.87% hydrocarbon: 20.10% termite: 18.30% ant_fungus_garden: 7.20% other: 6.18% wasp: 5.94% sediment: 4.86% mosquito: 3.30% ant: 2.37% wastewater: 1.80% gut: 1.65% marine: 1.32% freshwater: 1.12%
85% identity (33651 hits) hydrocarbon: 35.74% beetle: 9.89% termite: 8.39% ant_fungus_garden: 5.57% gut: 4.91% marine: 4.65% mosquito: 4.62% other: 3.83% sediment: 3.82% ant: 3.61% bioreactor: 3.44% food: 3.15% wasp: 2.83% wastewater: 2.78% soil: 1.59% biofilm: 1.18%
80% identity (61558 hits) hydrocarbon: 38.81% bioreactor: 11.73% beetle: 6.96% termite: 5.40% marine: 4.28% ant_fungus_garden: 3.63% other: 3.52% gut: 3.48% sediment: 3.38% mosquito: 3.21% food: 3.12% human: 2.89% ant: 2.69% wasp: 2.03% wastewater: 1.97% soil: 1.51% biofilm: 1.40%
