# Supplementary material for: MetaMetaDB: A Database and Analytic System for Investigating Microbial Habitability
Source: PLoS One. 2014 Jan 27;9(1):e87126. doi: 10.1371/journal.pone.0087126 (PMC3903645; doi:10.1371/journal.pone.0087126)
Supplement: Table S1 — Accession numbers of sequence run files used in MetaMetaDB. (PDF) [file pone.0087126.s003.pdf]

Table S1. Accession numbers of sequence run files used in MetaMetaDB.

**ant fungus garden metagenome**								
SRR124268	SRR206949	SRR206950	SRR206951	SRR357539	SRR357540	SRR357541	SRR357542	SRR357543
SRR357544	SRR357545	SRR357546	SRR357547	SRR357548	SRR357549	SRR357550	SRR357551	SRR357552
SRR357553	SRR357554	SRR357555	SRR357556	SRR357557	SRR357558	SRR357559	SRR357560	SRR357561
SRR357562	SRR357563	SRR357564	SRR357565	SRR357566	SRR357567	SRR357568	SRR357569	SRR357570
SRR357571	SRR357572	SRR357573	SRR357574	SRR357575	SRR357576	SRR357577	SRR357578	SRR357579
SRR357580	SRR357581	SRR357582	SRR357583	SRR357584	SRR357585	SRR357586	SRR357587	SRR357588
SRR357589	SRR357590	SRR357591	SRR357592	SRR357593	SRR357594	SRR357595	SRR357596	SRR357597
SRR357598	SRR357599	SRR357600	SRR357601	SRR357602	SRR357603	SRR357604	SRR357605	SRR357606
SRR357607	SRR357608	SRR357609	SRR357610	SRR357611	SRR357612	SRR357613	SRR357614	SRR357615
SRR357616	SRR357617	SRR357618	SRR357619	SRR357620	SRR357621	SRR357622	SRR357623	SRR357624
SRR357625	SRR357626	SRR357627	SRR357628	SRR357629	SRR357630	SRR357631	SRR357632	SRR357633
SRR357634	SRR357635	SRR357636	SRR357637	SRR360503	SRR357639	SRR357640	SRR357641	SRR357642
SRR357643	SRR357644	SRR357645	SRR357646	SRR357647	SRR357648	SRR357649	SRR357650	SRR357651
SRR357652	SRR357653	SRR357654	SRR357655	SRR357656	SRR357657	SRR357658	SRR357659	SRR357660
SRR357661								
**ant metagenome**								
SRR332520	SRR332521	SRR332522	SRR332523	SRR332524	SRR332525	SRR332526	SRR359017	SRR359018
**beach sand metagenome**								
ERR039826	ERR039827	ERR039828	ERR039829					
**beetle metagenome**								
SRR332527	SRR332528	SRR332529	SRR332530	SRR332531	SRR332532	SRR332533	SRR332534	SRR332535
SRR332536	SRR332537	SRR332539	SRR332540	SRR359003	SRR359004	SRR359005	SRR359006	SRR359007
SRR359008								
**biofilm metagenome**								
DRR001167	DRR001168	DRR001169	DRR001170	DRR001822	DRR001823	SRR071714	SRR071718	SRR071723
SRR071724	SRR071725	SRR071726	SRR071727	SRR071729	SRR071730	SRR071731	SRR071732	SRR071733
SRR071734	SRR071735	SRR071736	SRR071737	SRR071738	SRR071739	SRR071753	SRR071754	SRR071755
SRR071756	SRR071757							
**bioreactor metagenome**								
SRR330071	SRR330072	SRR330069	SRR343279	SRR343280	SRR343281	SRR369428	SRR369429	SRR369430
SRR369431	SRR369432	SRR369433	SRR369434	SRR369435	SRR369436	SRR369437	SRR369438	SRR369439
SRR369440	SRR369441	SRR369442	SRR369443	SRR369444	SRR371535	SRR371536	SRR371537	SRR371538
SRR371539	SRR371540	SRR371541	SRR371542	SRR371543	SRR371544	SRR371545	SRR371546	SRR371547
SRR371548	SRR371549	SRR371550						
**bioreactor sludge metagenome**								
SRR090227	SRR095857	SRR095858	SRR095859	SRR095860	SRR095861	SRR095862	SRR095863	SRR095864
SRR095865	SRR095866	SRR095867	SRR095868	SRR095869	SRR095870	SRR095871	SRR095872	SRR095873
SRR095874	SRR095875	SRR095876	SRR095877	SRR095878	SRR095879	SRR095880	SRR095881	SRR095882
SRR095883	SRR095884	SRR095885	SRR095886	SRR095887	SRR095888	SRR095889	SRR095890	SRR095891
SRR095892	SRR095893	SRR095894	SRR095895	SRR095896	SRR095897	SRR095898	SRR095899	SRR095900
SRR095901	SRR095902	SRR095903	SRR095904	SRR095905	SRR095906	SRR095907	SRR095908	SRR095909
SRR095910	SRR095911	SRR095912	SRR095913	SRR095914	SRR095915	SRR095916	SRR095917	SRR095918
SRR095919	SRR095920	SRR095921	SRR095922	SRR095923	SRR095924	SRR095925	SRR095926	SRR095927
SRR095928	SRR095929	SRR095930	SRR095931	SRR095932	SRR095933	SRR095934	SRR095935	SRR095936
SRR095937	SRR095938	SRR095939	SRR095940	SRR095941	SRR095942	SRR095943	SRR095944	SRR095945
SRR095946	SRR095947	SRR095948	SRR095949	SRR095950	SRR095951	SRR095952	SRR095953	SRR095954
SRR095955	SRR095956	SRR095957	SRR095958	SRR095959	SRR095960	SRR095961	SRR095962	SRR095963
SRR095964	SRR095965	SRR095966	SRR095967	SRR095968	SRR095970	SRR095971	SRR095972	SRR095973
SRR095974	SRR095975	SRR095976	SRR095977	SRR095978	SRR095979	SRR095980	SRR095981	SRR095982
SRR095983	SRR095984	SRR095985	SRR095986	SRR095987	SRR095988	SRR095989	SRR095990	SRR095991
SRR095992	SRR095993	SRR095994	SRR095995	SRR095996	SRR095997	SRR095998	SRR095999	SRR096000
SRR096001	SRR096002	SRR096003	SRR096004	SRR096005	SRR096006	SRR096007	SRR096008	SRR096009
SRR096010	SRR096011	SRR096012	SRR096013	SRR096014	SRR096015	SRR096016	SRR096017	SRR096018
SRR096019	SRR096020	SRR096021	SRR096022	SRR096023	SRR096024	SRR096025	SRR096026	SRR096027
SRR096028	SRR096029	SRR096030	SRR096031	SRR096032	SRR096033	SRR096034	SRR096035	SRR096036
SRR096037	SRR096038	SRR096039	SRR096040	SRR096041	SRR096042	SRR096043	SRR096044	SRR096045

SRR096046	SRR096047	SRR096048	SRR096049	SRR096050	SRR096051	SRR096052	SRR096053	SRR096054
SRR096055	SRR096056	SRR096057	SRR096058	SRR096059	SRR096060	SRR096061	SRR096062	SRR096063
SRR096064	SRR096065	SRR096066	SRR096067	SRR096068	SRR096069	SRR096070	SRR096071	SRR096072
SRR096073	SRR096074	SRR096075	SRR096076	SRR096077	SRR096078	SRR096079	SRR096080	SRR096081
SRR363442								
**bovine gut metagenome**								
ERR032057	ERR032062	SRR019239	SRR019240	SRR019241	SRR019242	SRR019243	SRR060160	SRR060161
SRR060162	SRR060163	SRR060158	SRR060159	SRR060164	SRR060165	SRR346426		
**chicken gut metagenome**								
ERR032059	SRR027046	SRR027047	SRR027048	SRR027049				
**compost metagenome**								
SRR121511	SRR121512	SRR121513	SRR121514	SRR121515	SRR121516	SRR121624	SRR121625	SRR121626
SRR121627	SRR121628	SRR121629	SRR121630	SRR121631	SRR121632	SRR121633	SRR121634	SRR121635
SRR121636	SRR121637	SRR121638	SRR121639	SRR121640	SRR124145			
**coral metagenome**								
SRR001052	SRR001077	SRR001078	SRR001079	SRR001080	SRR001081	SRR001082	SRR001083	SRR001084
SRR001085	SRR001086	SRR001087	SRR001088	SRR100549				
**fish metagenome**								
DRR001611	DRR001612	DRR001613	DRR001614	DRR001615	DRR001616	DRR001617	DRR001618	DRR001619
DRR001620	DRR001621	ERR012013	ERR012014	ERR012015	ERR042935	ERR042931	ERR042934	ERR042937
SRR001065	SRR001066	SRR001067	SRR001068	SRR001069	SRR001070	SRR001071	SRR001072	
**food fermentation metagenome**								
DRR003208								
**food metagenome**								
DRR001368	DRR001369	DRR001610	DRR001713	SRR098419	SRR256747	SRR256748	SRR256749	SRR256750
SRR256751	SRR256752	SRR256753	SRR256754	SRR256755	SRR256756			
**freshwater metagenome**								
DRR001609	ERR011367	ERR011374	ERR011375	ERR032063	ERR042932	SRR001047	SRR001064	SRR001073
SRR001074	SRR001075	SRR001076	SRR006906	SRR006907	SRR013515	SRR013516	SRR013517	SRR013518
SRR013519	SRR013520	SRR013521	SRR014834	SRR017899	SRR063691	SRR091234	SRR094470	SRR192306
SRR192307	SRR192321	SRR287655	SRR287665	SRR305966	SRR305967	SRR398144		
**freshwater sediment metagenome**								
DRR001608	ERR042933							
**groundwater metagenome**								
SRR400634								
**gut metagenome**								
ERR032064	SRR054690	SRR054692	SRR054693	SRR054694	SRR054695	SRR054696	SRR054697	SRR054698
SRR054699	SRR054700	SRR054701	SRR054702	SRR155572	SRR065387	SRR070954	SRR070998	SRR071063
SRR071069	SRR071072	SRR071103	SRR071105	SRR071109	SRR071229	SRR071291	SRR071292	SRR071293
SRR071294	SRR071295	SRR071296	SRR071298	SRR071299	SRR071300	SRR071301	SRR071302	SRR094070
SRR094069	SRR094071	SRR094073	SRR094074	SRR094072	SRR100123	SRR100172	SRR208165	SRR208166
SRR303350	SRR309121	SRR309122	SRR309123	SRR309124	SRR350759	SRR350760	SRR350761	SRR350762
SRR350763	SRR350764	SRR350765	SRR350766	SRR350767	SRR350768	SRR350769	SRR350770	SRR350771
SRR350772	SRR350773	SRR350774	SRR350775	SRR350776	SRR350777	SRR350778	SRR350779	SRR350780
SRR350781	SRR350782	SRR350783	SRR350784	SRR350785	SRR350786	SRR350787	SRR350788	SRR350789
SRR350790	SRR350791	SRR350792	SRR350793	SRR350794	SRR350795	SRR350796	SRR350797	SRR350798
**honeybee metagenome**								
DRR001869	DRR001870							
**hot springs metagenome**								
SRR074959	SRR076608	SRR074960	SRR076609	SRR364864	SRR364865	SRR364867	SRR364869	SRR364870
SRR364872	SRR364878	SRR364707	SRR364708					
**human gut metagenome**								
ERR011066	ERR011060	ERR011065	ERR011073	ERR011059	ERR011061	ERR011084	ERR011072	ERR011078
ERR011068	ERR011063	ERR011069	ERR011085	ERR011082	ERR011079	ERR011077	ERR011067	ERR011083
ERR011076	ERR011075	ERR011062	ERR011074	ERR011071	ERR011081	ERR011086	ERR011080	ERR011064
ERR011070	ERR011058	ERR022890	ERR022891	ERR022892	ERR022893	ERR022894	ERR022895	ERR032056
ERR032060	ERR037340	ERR037341	ERR037342	ERR037343	ERR037344	ERR037345	ERR037346	ERR037347
ERR037348	ERR037349	ERR037350	ERR037351	ERR037352	ERR037353	ERR037354	ERR037355	ERR037356
ERR037357	ERR037358	ERR037359	ERR037360	ERR037361	ERR037362	ERR037363	ERR037364	ERR037365
ERR037366	ERR037367	ERR037368	ERR037369	ERR037370	ERR037371	ERR037372	ERR037373	ERR037374

SRR001667	SRR001668	SRR001669	SRR001670	SRR004254	SRR004255	SRR004256	SRR004257	SRR004258
SRR029687	SRR029692	SRR029694	SRR029696	SRR029699	SRR029700	SRR029701	SRR029702	SRR029703
SRR029686	SRR029688	SRR029689	SRR029690	SRR029691	SRR029693	SRR029695	SRR029697	SRR029698
SRR005479	SRR006545	SRR006546	SRR006547	SRR006538	SRR006539	SRR006540	SRR006541	SRR006542
SRR006543	SRR006544	SRR017342	SRR017532	SRR017533	SRR017534	SRR017535	SRR017536	SRR017537
SRR017538	SRR017539	SRR017540	SRR017541	SRR017542	SRR017543	SRR017544	SRR017545	SRR017546
SRR017547	SRR017548	SRR017549	SRR017550	SRR017551	SRR017552	SRR017553	SRR017554	SRR017555
SRR017556	SRR017557	SRR017558	SRR017559	SRR017560	SRR017561	SRR017562	SRR017563	SRR017564
SRR017565	SRR017566	SRR017567	SRR017568	SRR020180	SRR029586	SRR029587	SRR029588	SRR029589
SRR029590	SRR029591	SRR029592	SRR029593	SRR029594	SRR029595	SRR029596	SRR029597	SRR029598
SRR029599	SRR029600	SRR029601	SRR029602	SRR029603	SRR040449	SRR040822	SRR068049	SRR068050
SRR068051	SRR068052	SRR068053	SRR068054	SRR068055	SRR068056	SRR068057	SRR068058	SRR068059
SRR068060	SRR068061	SRR068062	SRR068063	SRR068064	SRR068065	SRR068066	SRR068067	SRR068068
SRR068069	SRR068070	SRR068071	SRR068072	SRR068073	SRR068074	SRR068075	SRR068076	SRR068077
SRR068078	SRR068079	SRR068080	SRR068081	SRR068082	SRR068083	SRR068084	SRR068085	SRR068086
SRR068087	SRR068088	SRR068089	SRR068090	SRR068091	SRR068092	SRR068093	SRR068094	SRR068095
SRR068096	SRR068097	SRR068098	SRR068099	SRR068100	SRR068101	SRR068102	SRR068103	SRR068104
SRR068105	SRR068106	SRR068107	SRR068108	SRR068109	SRR069226	SRR068116	SRR068117	SRR068121
SRR068112	SRR068118	SRR068119	SRR068120	SRR068113	SRR068114	SRR068115	SRR068111	SRR073551
SRR073552	SRR073554	SRR073557	SRR360587					
**human metagenome**								
ERR006599	ERR006600	ERR006601	ERR006602	ERR006603	ERR006604	ERR006605	ERR006606	ERR006607
ERR006608	ERR006609	ERR006610	ERR006611	ERR006612	ERR006613	ERR006614	ERR006615	ERR006616
ERR006617	ERR006618	ERR006619	ERR006620	ERR006621	ERR006622	ERR006623	ERR006624	ERR006625
ERR006626	ERR006627	ERR006628	ERR006629	ERR006630	ERR006631	ERR006632	ERR006633	ERR006634
ERR006635	ERR006636	ERR006637	ERR006638	ERR006639	ERR006640	ERR006641	ERR006642	ERR006643
ERR006644	ERR006645	ERR006646	ERR006647	ERR006648	ERR006649	ERR006650	ERR006651	ERR006652
ERR006653	ERR006654	ERR006655	ERR006656	ERR006657	ERR006658	ERR006659	ERR006660	ERR006661
ERR006662	ERR006663	ERR006664	ERR006665	ERR006666	ERR006667	ERR006668	ERR006669	ERR006670
ERR006671	ERR006672	ERR006673	ERR006674	ERR006675	ERR006676	ERR006677	ERR006678	ERR006679
ERR006680	ERR006681	ERR006682	ERR006683	ERR006684	ERR006685	ERR006686	ERR006687	ERR006688
ERR006689	ERR006690	ERR006691	ERR006692	ERR006693	ERR006694	ERR006695	ERR006696	ERR006697
ERR006698	ERR006699	ERR006700	ERR006701	ERR006702	ERR006703	ERR006704	ERR006705	ERR006706
ERR006707	ERR006708	ERR006709	ERR006710	ERR006711	ERR006712	ERR006713	ERR006714	ERR006715
ERR006716	ERR006717	ERR006718	ERR006719	ERR006720	ERR006721	ERR006722	ERR006723	ERR006724
ERR006725	ERR006726	ERR006727	ERR006728	ERR006729	ERR006730	ERR006731	ERR006732	ERR006733
ERR006734	ERR006735	ERR006736	ERR006737	ERR006738	ERR006739	ERR006740	ERR006741	ERR006742
ERR006743	ERR006744	ERR006745	ERR006746	ERR006747	ERR006748	ERR006749	ERR006750	ERR006751
ERR006752	ERR006753	ERR006754	ERR006755	ERR006756	ERR006757	ERR006758	ERR006759	ERR006760
ERR006761	ERR006762	ERR006763	ERR006764	ERR006765	ERR006766	ERR006767	ERR006768	ERR006769
ERR006770	ERR006771	ERR006772	ERR006773	ERR006774	ERR006775	ERR006776	ERR006777	ERR006778
ERR006779	ERR006780	ERR006781	ERR006782	ERR006783	ERR006784	ERR006785	ERR006786	ERR006787
ERR006788	ERR006789	ERR006790	ERR006791	ERR006792	ERR006793	ERR006794	ERR006795	ERR006796
ERR006797	ERR006798	ERR006799	ERR006800	ERR006801	ERR006802	ERR006803	ERR006804	ERR006805
ERR006806	ERR006807	ERR006808	ERR006809	ERR006810	ERR006811	ERR006812	ERR006813	ERR006814
ERR006815	ERR006816	ERR006817	ERR006818	ERR006819	ERR006820	ERR006821	ERR006822	ERR006823
ERR006824	ERR006825	ERR006826	ERR006827	ERR006828	ERR006829	ERR006830	ERR006831	ERR006832
ERR006833	ERR006834	ERR006835	ERR006836	ERR006837	ERR006838	ERR006839	ERR006840	ERR006841
ERR006842	ERR006843	ERR006844	ERR006845	ERR006846	ERR006847	ERR006848	ERR006849	ERR006850
ERR006851	ERR006852	ERR006853	ERR006854	ERR006855	ERR006856	ERR006857	ERR006858	ERR006859
ERR006860	ERR006861	ERR006862	ERR006863	ERR006864	ERR006865	ERR006866	ERR006867	ERR006868
ERR006869	ERR006870	ERR006871	ERR006872	ERR006873	ERR006874	ERR006875	ERR006876	ERR006877
ERR006878	ERR006879	ERR006880	ERR006881	ERR006882	ERR006883	ERR006884	ERR006885	ERR006886
ERR006887	ERR006888	ERR006889	ERR006890	ERR006891	ERR006892	ERR006893	ERR006894	ERR006895
ERR006896	ERR006897	ERR006898	ERR006899	ERR006900	ERR006901	ERR006902	ERR006903	ERR006904
ERR006905	ERR006906	ERR006907	ERR006908	ERR006909	ERR006910	ERR006911	ERR006912	ERR006913
ERR006914	ERR006915	ERR006916	ERR006917	ERR006918	ERR006919	ERR006920	ERR006921	ERR006922
ERR006923	ERR006924	ERR006925	ERR006926	ERR006927	ERR006928	ERR006929	ERR006930	ERR006931
ERR006932	ERR006933	ERR006934	ERR006935	ERR006936	ERR006937	ERR006938	ERR006939	ERR006940
ERR006941	ERR006942	ERR006943	ERR006944	ERR006945	ERR006946	ERR006947	ERR006948	ERR006949
ERR006950	ERR006951	ERR006952	ERR006953	ERR006954	ERR006955	ERR006956	ERR006957	ERR006958

ERR006959	ERR006960	ERR006961	ERR006962	ERR006963	ERR006964	ERR006965	ERR006966	ERR006967
ERR006968	ERR006969	ERR006970	ERR006971	ERR006972	ERR006973	ERR006974	ERR006975	ERR006976
ERR006977	ERR006978	ERR006979	ERR006980	ERR006981	ERR006982	ERR006983	ERR006984	ERR006985
ERR006986	ERR006987	ERR006988	ERR006989	ERR006990	ERR006991	ERR006992	ERR006993	ERR006994
ERR006995	ERR006996	ERR006997	ERR006998	ERR006999	ERR007000	ERR007001	ERR007002	ERR007003
ERR007004	ERR007005	ERR007006	ERR007007	ERR007008	ERR007009	ERR007010	ERR007011	ERR007012
ERR007013	ERR007014	ERR007015	ERR007016	ERR007017	ERR007018	ERR007019	ERR007020	ERR007021
ERR007022	ERR007023	ERR007024	ERR007025	ERR007026	ERR007027	ERR007028	ERR007029	ERR007030
ERR007031	ERR007032	ERR007033	ERR007034	ERR007035	ERR007036	ERR007037	ERR007038	ERR007039
ERR007040	ERR007041	ERR007042	ERR007043	ERR007044	ERR007045	ERR007046	ERR007047	ERR007048
ERR007049	ERR007050	ERR007051	ERR007052	ERR007053	ERR007054	ERR007055	ERR007056	ERR007057
ERR007058	ERR007059	ERR007060	ERR007061	ERR007062	ERR007063	ERR007064	ERR007065	ERR007066
ERR007067	ERR007068	ERR007069	ERR007070	ERR007071	ERR007072	ERR007073	ERR007074	ERR007075
ERR007076	ERR007077	ERR007078	ERR007079	ERR007080	ERR007081	ERR007082	ERR007083	ERR007084
ERR007085	ERR007086	ERR007087	ERR007088	ERR007089	ERR007090	ERR007091	ERR007092	ERR007093
ERR007094	ERR007095	ERR007096	ERR007097	ERR007098	ERR007099	ERR007100	ERR007101	ERR007102
ERR007103	ERR007104	ERR007105	ERR007106	ERR007107	ERR007108	ERR007109	ERR007110	ERR007111
ERR007112	ERR007113	ERR007114	ERR007115	ERR007116	ERR007117	ERR007118	ERR007119	ERR007120
ERR007121	ERR007122	ERR007123	ERR007124	ERR007125	ERR007126	ERR007127	ERR007128	ERR007129
ERR007130	ERR007131	ERR007132	ERR007133	ERR007134	ERR007135	ERR007136	ERR007137	ERR007138
ERR007139	ERR007140	ERR007141	ERR007142	ERR007143	ERR007144	ERR007145	ERR007146	ERR007147
ERR007148	ERR007149	ERR007150	ERR007151	ERR007152	ERR007153	ERR007154	ERR007155	ERR007156
ERR007157	ERR007158	ERR007159	ERR007160	ERR007161	ERR007162	ERR007163	ERR007164	ERR007165
ERR007166	ERR007167	ERR007168	ERR007169	ERR007170	ERR007171	ERR007172	ERR007173	ERR007174
ERR007175	ERR007176	ERR007177	ERR007178	ERR007179	ERR007180	ERR007181	ERR007182	ERR007183
ERR007184	ERR007185	ERR007186	ERR007187	ERR007188	ERR007189	ERR007190	ERR007191	ERR007192
ERR007193	ERR007194	ERR007195	ERR007196	ERR007197	ERR007198	ERR007199	ERR007200	ERR007201
ERR007202	ERR007203	ERR007204	ERR007205	ERR007206	ERR007207	ERR007208	ERR007209	ERR007210
ERR007211	ERR007212	ERR007213	ERR007214	ERR007215	ERR007216	ERR007217	ERR007218	ERR007219
ERR007220	ERR007221	ERR007222	ERR007223	ERR007224	ERR007225	ERR007226	ERR007227	ERR007228
ERR007229	ERR007230	ERR007231	ERR007232	ERR007233	ERR007234	ERR007235	ERR007236	ERR007237
ERR007238	ERR007239	ERR007240	ERR007241	ERR007242	ERR007243	ERR007244	ERR007245	ERR007246
ERR007247	ERR007248	ERR007249	ERR007250	ERR007251	ERR007252	ERR007253	ERR007254	ERR007255
ERR007256	ERR007257	ERR007258	ERR007259	ERR007260	ERR007261	ERR007262	ERR007263	ERR007264
ERR007265	ERR007266	ERR007267	ERR007268	ERR007269	ERR007270	ERR007271	ERR007272	ERR007273
ERR007274	ERR007275	ERR007276	ERR007277	ERR007278	ERR007279	ERR007280	ERR007281	ERR007282
ERR007283	ERR007284	ERR007285	ERR007286	ERR007287	ERR007288	ERR007289	ERR007290	ERR007291
ERR007292	ERR007293	ERR007294	ERR007295	ERR007296	ERR007297	ERR007298	ERR007299	ERR007300
ERR007301	ERR007302	ERR007303	ERR007304	ERR007305	ERR007306	ERR007307	ERR007308	ERR007309
ERR007310	ERR007311	ERR007312	ERR007313	ERR007314	ERR007315	ERR007316	ERR007317	ERR007318
ERR007319	ERR007320	ERR007321	ERR007322	ERR007323	ERR007324	ERR007325	ERR007326	ERR007327
ERR007328	ERR007329	ERR007330	ERR007331	ERR007332	ERR007333	ERR007334	ERR007335	ERR007336
ERR007337	ERR007338	ERR007339	ERR007340	ERR007341	ERR007342	ERR007343	ERR007344	ERR007345
ERR007346	ERR007347	ERR007348	ERR007349	ERR007350	ERR007351	ERR007352	ERR007353	ERR007354
ERR007355	ERR007356	ERR007357	ERR007358	ERR007359	ERR007360	ERR007361	ERR007362	ERR007363
ERR007364	ERR007365	ERR007366	ERR007367	ERR007368	ERR007369	ERR007370	ERR007371	ERR007372
ERR007373	ERR007374	ERR007375	ERR007376	ERR007377	ERR007378	ERR007379	ERR007380	ERR007381
ERR007382	ERR007383	ERR007384	ERR007385	ERR007386	ERR007387	ERR007388	ERR007389	ERR007390
ERR007391	ERR007392	ERR007393	ERR007394	ERR007395	ERR007396	ERR007397	ERR007398	ERR007399
ERR007400	ERR007401	ERR007402	ERR007403	ERR007404	ERR007405	ERR007406	ERR007407	ERR007408
ERR007409	ERR007410	ERR007411	ERR007412	ERR007413	ERR049777	ERR049778	ERR049779	ERR049780
ERR049781	ERR049782	ERR049783	ERR049784	ERR049785	ERR049786	SRR028690	SRR028691	SRR028692
SRR028693	SRR028694	SRR028695	SRR028696	SRR028697	SRR028698	SRR028699	SRR028700	SRR028701
SRR028702	SRR028703	SRR028704	SRR028705	SRR028706	SRR028707	SRR028708	SRR028709	SRR028710
SRR028711	SRR028712	SRR028713	SRR028714	SRR028715	SRR028716	SRR028717	SRR028718	SRR029122
SRR062670	SRR062671	SRR062672	SRR062673	SRR062674	SRR062675	SRR062676	SRR062677	SRR062678
SRR062679	SRR062680	SRR062681	SRR062682	SRR062683	SRR062684	SRR062685	SRR062686	SRR062687
SRR062688	SRR062689	SRR062690	SRR062691	SRR062692	SRR062693	SRR062694	SRR062695	SRR062696
SRR062697	SRR062698	SRR062699	SRR062700	SRR062701	SRR062702	SRR062703	SRR062704	SRR062705
SRR062706	SRR062707	SRR062708	SRR062709	SRR062710	SRR062711	SRR062712	SRR062713	SRR062714
SRR062715	SRR062716	SRR062717	SRR062718	SRR062719	SRR062720	SRR062721	SRR062722	SRR062723

SRR062724	SRR062725	SRR062726	SRR062727	SRR062728	SRR062729	SRR062730	SRR062731	SRR062732
SRR062733	SRR062734	SRR062735	SRR062736	SRR062737	SRR062738	SRR062739	SRR062740	SRR062741
SRR062742	SRR062743	SRR062744	SRR062745	SRR062746	SRR062747	SRR062748	SRR062749	SRR062750
SRR062751	SRR062752	SRR062753	SRR062755	SRR062756	SRR062757	SRR062758	SRR062759	SRR062760
SRR062761	SRR062762	SRR062763	SRR062764	SRR062765	SRR062766	SRR062767	SRR062768	SRR062769
SRR062770	SRR062771	SRR062772	SRR062773	SRR062774	SRR062775	SRR062776	SRR062777	SRR062778
SRR062779	SRR062780	SRR062781	SRR062782	SRR062783	SRR062784	SRR062785	SRR062786	SRR062787
SRR062788	SRR062789	SRR062790	SRR062791	SRR062792	SRR062793	SRR062794	SRR062795	SRR062796
SRR062797	SRR062798	SRR062799	SRR062800	SRR062801	SRR062802	SRR062803	SRR062804	SRR062805
SRR062806	SRR062807	SRR062808	SRR062809	SRR062810	SRR062811	SRR062812	SRR062813	SRR062814
SRR062815	SRR062816	SRR062817	SRR062818	SRR062819	SRR062820	SRR062821	SRR062822	SRR062823
SRR062824	SRR062825	SRR062826	SRR062827	SRR062828	SRR062829	SRR062830	SRR062831	SRR062832
SRR062833	SRR062834	SRR062835	SRR062836	SRR062837	SRR062838	SRR062839	SRR062840	SRR062842
SRR062843	SRR062844	SRR062845	SRR062846	SRR062847	SRR062848	SRR062849	SRR062850	SRR062851
SRR062852	SRR062853	SRR062854	SRR062855	SRR062856	SRR062857	SRR062858	SRR062859	SRR062860
SRR062861	SRR062862	SRR062863	SRR062864	SRR062865	SRR062866	SRR062867	SRR062868	SRR062869
SRR062870	SRR062871	SRR062872	SRR062873	SRR062874	SRR062875	SRR062876	SRR062877	SRR062878
SRR062879	SRR062880	SRR062881	SRR062882	SRR062883	SRR062884	SRR062885	SRR062886	SRR062887
SRR062888	SRR062889	SRR062890	SRR062891	SRR062892	SRR062893	SRR062894	SRR062895	SRR062896
SRR062897	SRR062898	SRR062899	SRR062900	SRR062901	SRR062902	SRR062903	SRR062904	SRR062905
SRR062906	SRR062907	SRR062908	SRR062909	SRR062910	SRR062911	SRR062912	SRR062913	SRR062914
SRR062915	SRR062916	SRR062917	SRR062918	SRR062919	SRR062920	SRR062921	SRR062922	SRR062923
SRR062924	SRR062925	SRR062926	SRR062927	SRR062928	SRR062929	SRR062930	SRR062931	SRR062932
SRR062934	SRR062935	SRR062936	SRR062937	SRR062938	SRR062939	SRR062940	SRR062941	SRR062942
SRR062943	SRR062944	SRR062945	SRR062946	SRR062947	SRR062948	SRR062949	SRR062950	SRR062951
SRR062952	SRR062953	SRR062954	SRR062955	SRR062956	SRR062957	SRR062958	SRR062959	SRR062960
SRR062961	SRR062962	SRR062963	SRR062964	SRR062965	SRR062966	SRR062967	SRR062968	SRR062969
SRR062970	SRR062971	SRR062972	SRR062973	SRR062974	SRR062975	SRR062976	SRR062977	SRR062978
SRR062979	SRR062980	SRR062981	SRR062982	SRR062983	SRR062984	SRR062985	SRR062986	SRR062987
SRR062988	SRR062989	SRR062990	SRR062992	SRR062993	SRR062994	SRR062995	SRR062996	SRR062997
SRR062998	SRR062999	SRR063000	SRR063001	SRR063002	SRR063003	SRR063004	SRR063005	SRR063006
SRR063007	SRR063008	SRR063009	SRR063010	SRR063011	SRR063012	SRR063013	SRR063014	SRR063015
SRR063016	SRR063017	SRR063018	SRR063019	SRR063020	SRR063021	SRR063022	SRR063023	SRR063024
SRR063025	SRR063026	SRR063027	SRR063028	SRR063029	SRR063030	SRR063031	SRR063032	SRR063033
SRR063034	SRR063035	SRR063036	SRR063037	SRR063038	SRR063039	SRR063040	SRR063041	SRR063042
SRR063043	SRR063044	SRR063045	SRR063046	SRR063047	SRR063048	SRR063049	SRR063050	SRR063051
SRR063052	SRR063053	SRR063054	SRR063055	SRR063056	SRR063057	SRR063058	SRR063059	SRR063060
SRR063061	SRR063062	SRR063063	SRR063064	SRR063065	SRR063066	SRR063067	SRR077426	SRR086165
SRR086166	SRR106308	SRR106819	SRR341547	SRR341953	SRR341954	SRR341955	SRR341956	SRR341957
SRR384890	SRR384891	SRR384892	SRR384893	SRR387542	SRR387543	SRR387544	SRR387545	SRR387546
SRR387547	SRR387548	SRR387549						
**human oral metagenome**								
SRR116771	SRR116769	SRR116770	SRR346809	SRR346810	SRR346811	SRR346812	SRR346813	SRR346814
SRR346815	SRR346816	SRR346817	SRR346818	SRR346819	SRR346820	SRR346821	SRR346822	SRR346823
SRR346824	SRR346825	SRR346826	SRR346827	SRR346828	SRR346829	SRR346830	SRR346831	SRR346832
SRR346833	SRR346834	SRR346835	SRR346836	SRR346837	SRR346838	SRR346839	SRR346840	SRR346841
SRR346842	SRR346843	SRR346844	SRR346845	SRR346846	SRR346847	SRR346848	SRR346849	SRR346850
SRR346851	SRR346852	SRR346853	SRR346854	SRR346855	SRR346856	SRR346857	SRR346858	SRR346859
SRR346860	SRR346861	SRR346862	SRR346863	SRR346864	SRR346865	SRR346866	SRR346867	SRR346868
SRR346869	SRR346870	SRR346871	SRR346872	SRR346873	SRR346874	SRR346875	SRR346876	SRR346877
SRR346878	SRR346879	SRR346880	SRR346881	SRR346882	SRR346883	SRR346884	SRR387669	SRR387670
SRR387937	SRR387938	SRR387939	SRR387940	SRR387941	SRR387942	SRR387943	SRR387944	SRR387945
SRR387946	SRR387947	SRR387948	SRR387949	SRR387950	SRR387951	SRR387952	SRR387953	SRR387954
SRR387955	SRR387956	SRR387957	SRR387958	SRR387959	SRR387960	SRR387961	SRR387962	SRR387963
SRR387964	SRR387965	SRR387966	SRR387967	SRR387968	SRR387969	SRR387970	SRR387971	SRR387972
SRR387973	SRR387974	SRR387975	SRR387976	SRR387977	SRR387978	SRR387979	SRR387980	SRR387981
SRR387982	SRR387983	SRR387984	SRR387985	SRR387986	SRR387987	SRR387988	SRR387989	SRR387990
SRR387991	SRR387992	SRR387993	SRR387994	SRR387995	SRR387996	SRR387997	SRR387998	SRR387999
SRR388000	SRR388001	SRR388002	SRR388003	SRR388004	SRR388005	SRR388006	SRR388007	SRR388008
SRR388009	SRR388010	SRR388011	SRR388012	SRR388013	SRR388014	SRR388015	SRR388016	SRR388017
SRR388018	SRR388019	SRR388020	SRR388021	SRR388022	SRR388023	SRR388024	SRR388025	SRR388026

SRR388027	SRR388028	SRR388029	SRR388030	SRR388031	SRR388032	SRR388033	SRR388034	SRR388035
SRR388036	SRR388037	SRR388038	SRR388039	SRR388040	SRR388041	SRR388042	SRR388043	SRR388044
SRR388045	SRR388046	SRR388047	SRR388048	SRR388049	SRR388050	SRR388051	SRR388052	SRR388053
SRR388054	SRR388055	SRR388056	SRR388057	SRR388058	SRR388059	SRR388060	SRR388061	SRR388062
SRR388063	SRR388064	SRR388065	SRR388066	SRR388067	SRR388068	SRR388069	SRR388070	SRR388071
SRR388072	SRR388073	SRR388074	SRR388075	SRR388076	SRR388077	SRR388078	SRR388079	SRR388080
SRR388081	SRR388082	SRR388083	SRR388084	SRR388085	SRR388086	SRR388087	SRR388088	SRR388089
SRR388090	SRR388091	SRR388092	SRR388093	SRR388094	SRR388095	SRR388096	SRR388097	SRR388098
SRR388099	SRR388100	SRR388101	SRR388102	SRR388103	SRR388104	SRR388105	SRR388106	SRR388107
SRR388108	SRR388109	SRR388110	SRR388111	SRR388112				
**human skin metagenome**								
DRR003662	DRR003663	DRR003664	DRR003665	DRR003666	DRR003667	DRR003668	DRR003669	DRR003670
DRR003671	DRR003672	DRR003673	DRR003674	DRR003675	DRR003676	DRR003677	DRR003678	DRR003679
DRR003680	DRR003681	DRR003682	DRR003683	DRR003684	DRR003685	SRR006061	SRR031989	SRR068450
SRR068451	SRR068452	SRR068453	SRR068454	SRR068455	SRR068456	SRR068457	SRR068458	SRR068459
SRR068460	SRR068461	SRR068462	SRR068463	SRR068464	SRR068465	SRR068466	SRR068467	SRR068468
SRR068469	SRR068470	SRR068471	SRR068472	SRR068473	SRR068474	SRR068475	SRR068476	SRR068477
SRR068478	SRR068479	SRR068480	SRR068481	SRR068482	SRR068483	SRR068484	SRR068485	SRR068486
SRR068487	SRR068488	SRR068489	SRR068490	SRR068491	SRR068492	SRR068493	SRR068494	SRR068495
SRR068496	SRR068497	SRR068498	SRR068499	SRR068500	SRR068501	SRR068502	SRR068503	SRR068504
SRR068505	SRR068506	SRR068507	SRR068508	SRR068509	SRR068510	SRR068511	SRR068512	SRR068513
SRR068514	SRR068515	SRR068516	SRR068517	SRR068518	SRR068519	SRR068520	SRR068521	SRR068522
SRR068523	SRR068524	SRR068525	SRR068526	SRR068527	SRR068528	SRR068529	SRR068530	SRR068531
SRR068532	SRR068533	SRR068534	SRR068535	SRR068536	SRR068537	SRR068538	SRR068539	SRR068540
SRR068541	SRR068542	SRR068543	SRR091767	SRR091768	SRR091769	SRR091770	SRR091771	SRR091772
SRR091773	SRR091774	SRR091775	SRR091776	SRR091777	SRR091778	SRR091779	SRR091780	SRR091781
SRR091782	SRR091783	SRR091784	SRR091785	SRR091786	SRR091787	SRR091788	SRR091789	SRR091790
SRR091791	SRR091792	SRR091793	SRR091794	SRR091795	SRR091796	SRR091797	SRR097824	SRR097825
SRR097826	SRR097827	SRR097828	SRR097829	SRR097830	SRR097831	SRR097832	SRR097833	SRR097834
SRR097835	SRR097836	SRR097837	SRR097838	SRR097839	SRR097840	SRR097841	SRR097842	SRR097843
SRR097844	SRR097845							
**hydrocarbon metagenome**								
SRR088835	SRR088837	SRR088839	SRR088840	SRR088842	SRR088844	SRR088845	SRR088846	SRR088847
SRR088848	SRR088850	SRR088852	SRR088854	SRR088855	SRR090140	SRR090141	SRR090143	SRR090145
SRR090146	SRR090147	SRR090148	SRR090149	SRR090150	SRR090157	SRR090160	SRR090161	SRR090162
SRR090163	SRR090164	SRR090165	SRR090166	SRR090167	SRR090168	SRR090169	SRR090170	SRR090171
SRR090172	SRR090173	SRR090174	SRR090175	SRR090176	SRR090177	SRR090178	SRR090179	SRR090180
SRR090181	SRR090182	SRR090183	SRR090184	SRR090185	SRR090186	SRR090187	SRR090188	SRR090189
SRR090190	SRR090191	SRR090192	SRR090193	SRR090194	SRR090195	SRR090196	SRR090197	SRR090199
SRR090200	SRR090201	SRR090202	SRR090203	SRR090204	SRR090205	SRR090207	SRR090208	SRR090260
SRR090261	SRR090262	SRR090263	SRR090264	SRR090265	SRR090266	SRR090267	SRR090268	SRR090270
SRR090274	SRR090276	SRR090277	SRR090278	SRR090279	SRR090280	SRR090281	SRR090282	SRR090283
SRR090284	SRR090285	SRR090286	SRR090287	SRR090288	SRR090289	SRR090290	SRR090291	SRR090292
SRR090293	SRR090294	SRR090295	SRR090296	SRR090297	SRR090298	SRR090299	SRR090300	SRR090365
SRR090367	SRR090368	SRR090369	SRR090370	SRR098445	SRR098458	SRR090371	SRR090372	SRR090374
SRR090375	SRR090376	SRR090377	SRR090378	SRR090379	SRR090380	SRR090381	SRR090382	SRR090383
SRR090384	SRR090385	SRR090386	SRR090387	SRR090388	SRR090389	SRR090390	SRR090391	SRR090392
SRR090393	SRR090394	SRR090395	SRR090396	SRR090397	SRR090398	SRR090400	SRR090401	SRR090402
SRR090403	SRR090404	SRR090405	SRR090406	SRR090407	SRR090408	SRR090409	SRR090410	SRR090411
SRR090412	SRR090413	SRR090414	SRR090415	SRR090416	SRR090417	SRR090418	SRR090419	SRR090420
SRR090421	SRR090422	SRR090427	SRR090428	SRR090429	SRR090430	SRR090431	SRR090432	SRR090433
SRR090434	SRR090435	SRR090436	SRR090437	SRR090482	SRR090483	SRR090484	SRR090485	SRR090486
SRR090487	SRR090488	SRR090536	SRR090537	SRR090538	SRR090539	SRR090540	SRR090541	SRR090542
SRR090543	SRR090544	SRR090545	SRR090546	SRR090547	SRR090548	SRR090549	SRR090550	SRR090551
SRR090552	SRR090553	SRR090554	SRR090555	SRR090556	SRR090557	SRR090558	SRR090559	SRR090560
SRR090561	SRR090562	SRR090564	SRR090565	SRR090566	SRR090567	SRR090568	SRR090569	SRR090610
SRR090611	SRR090612	SRR090613	SRR090614	SRR090615	SRR090616	SRR090617	SRR090618	SRR090620
SRR090621	SRR090622	SRR090623	SRR090624	SRR090626	SRR090627	SRR090628	SRR090629	SRR090630
SRR090631	SRR090632	SRR090633	SRR090635	SRR090640	SRR090641	SRR090642	SRR090644	SRR090645
SRR090646	SRR090647	SRR090648	SRR090649	SRR090650	SRR090651	SRR090652	SRR090653	SRR090655
SRR090656	SRR090657	SRR090658	SRR090659	SRR090660	SRR090661	SRR090662	SRR090663	SRR090664

SRR090665	SRR090666	SRR090667	SRR097652	SRR097657	SRR097658	SRR097659	SRR097660	SRR097661
SRR097712	SRR097662	SRR097716	SRR097663	SRR097717	SRR097664	SRR097704	SRR097665	SRR097718
SRR097666	SRR097719	SRR097667	SRR099838	SRR097668	SRR097710	SRR097669	SRR099839	SRR097670
SRR097671	SRR097672	SRR097675	SRR097676	SRR097679	SRR097680	SRR097683	SRR097686	SRR097687
SRR097690	SRR097691	SRR097713	SRR097694	SRR097697	SRR098446	SRR098459	SRR098447	SRR098460
SRR098448	SRR098461	SRR098449	SRR098462	SRR098450	SRR098463	SRR098451	SRR098464	SRR098452
SRR098465	SRR098453	SRR098466	SRR098454	SRR098467	SRR098455	SRR098468	SRR098456	SRR098469
SRR098457	SRR098470	SRR098704	SRR098705	SRR098706	SRR098707	SRR098708	SRR098709	SRR098710
SRR098711	SRR098712	SRR098713	SRR098714	SRR098715	SRR098716	SRR098717	SRR098718	SRR098719
SRR098720	SRR098721	SRR098722	SRR098723	SRR098947	SRR098948	SRR098949	SRR098950	SRR098999
SRR099001	SRR099003	SRR099015	SRR099017	SRR099019	SRR099021	SRR099023	SRR099024	SRR099025
SRR099026	SRR099027	SRR099028	SRR099029	SRR099030	SRR099032	SRR099044	SRR099045	SRR099046
SRR099047	SRR099048	SRR099049	SRR099050	SRR099057	SRR099058	SRR099059	SRR099060	SRR099061
SRR099062	SRR099063	SRR099065	SRR099066	SRR099067	SRR099068	SRR099069	SRR099070	SRR099071
SRR099072	SRR099073	SRR099074	SRR099077	SRR099078	SRR099079	SRR099080	SRR099081	SRR099082
SRR099083	SRR099084	SRR099085	SRR099086	SRR099089	SRR099090	SRR099091	SRR099092	SRR099093
SRR099094	SRR099095	SRR099096	SRR099097	SRR099098	SRR099099	SRR099100	SRR099101	SRR099104
SRR099105	SRR099136	SRR099137	SRR099138	SRR099139	SRR099140	SRR099154	SRR099155	SRR099156
SRR099157	SRR099158	SRR099159	SRR099160	SRR099161	SRR099162	SRR099163	SRR099164	SRR099165
SRR099166	SRR099167	SRR099168	SRR099169	SRR099170	SRR099171	SRR099172	SRR099173	SRR099174
SRR099175	SRR099176	SRR099177	SRR099178	SRR099179	SRR099180	SRR099181	SRR099182	SRR099183
SRR099184	SRR099185	SRR099186	SRR099187	SRR099188	SRR099189	SRR099190	SRR099191	SRR099192
SRR099193	SRR099194	SRR099195	SRR099196	SRR099197	SRR099198	SRR099199	SRR099200	SRR099201
SRR099202	SRR099203	SRR099204	SRR099205	SRR099390	SRR099391	SRR099392	SRR099393	SRR099394
SRR099395	SRR099396	SRR099397	SRR099398	SRR099399	SRR099400	SRR099401	SRR099402	SRR099403
SRR099404	SRR099409	SRR346427	SRR346434	SRR346441	SRR346444	SRR346445	SRR346448	SRR346449
SRR346450	SRR346451	SRR346452	SRR346453	SRR346454	SRR351323	SRR352728	SRR389312	SRR389313
SRR389314	SRR389315							
**hydrothermal vent metagenome**								
SRR029255								
**hypersaline lake metagenome**								
SRR364697	SRR364698	SRR364699	SRR364700	SRR364701				
**ice metagenome**								
SRR002326	SRR002327	SRR002328						
**marine metagenome**								
ERR011357	ERR011363	ERR011356	ERR011361	ERR011365	ERR011362	ERR011364	ERR011366	ERR011358
ERR011360	ERR011359	SRR000281	SRR000282	SRR000283	SRR000284	SRR000285	SRR000286	SRR000287
SRR000288	SRR000672	SRR000673	SRR000674	SRR000675	SRR000676	SRR000677	SRR000678	SRR000679
SRR000680	SRR000681	SRR000682	SRR000683	SRR000684	SRR000685	SRR000686	SRR000687	SRR000688
SRR000689	SRR000690	SRR000691	SRR000692	SRR000693	SRR000694	SRR000695	SRR000696	SRR000697
SRR000698	SRR000699	SRR000700	SRR000701	SRR000905	SRR000906	SRR000907	SRR001034	SRR001035
SRR001036	SRR001037	SRR001038	SRR001039	SRR001040	SRR001041	SRR001042	SRR001048	SRR001049
SRR001050	SRR001051	SRR001663	SRR016610	SRR017883	SRR019190	SRR019554	SRR019192	SRR019191
SRR019193	SRR019196	SRR019195	SRR019194	SRR019197	SRR019198	SRR019199	SRR019200	SRR019201
SRR019202	SRR019188	SRR019189	SRR019203	SRR019204	SRR020514	SRR023397	SRR023398	SRR023401
SRR023765	SRR023766	SRR023767	SRR023768	SRR023769	SRR023770	SRR023772	SRR023773	SRR023775
SRR023776	SRR023777	SRR023778	SRR023774	SRR027044	SRR027045	SRR027086	SRR027087	SRR027088
SRR027089	SRR027090	SRR027091	SRR027092	SRR027093	SRR027094	SRR027095	SRR027193	SRR027194
SRR027195	SRR027196	SRR027197	SRR027198	SRR027199	SRR027200	SRR027201	SRR027202	SRR027203
SRR027204	SRR027207	SRR027208	SRR027209	SRR027210	SRR027211	SRR027212	SRR027213	SRR027214
SRR027215	SRR027216	SRR027217	SRR027218	SRR027219	SRR027220	SRR027221	SRR027222	SRR027223
SRR027224	SRR027225	SRR027226	SRR027227	SRR027228	SRR027229	SRR027230	SRR027231	SRR027232
SRR027233	SRR027234	SRR027235	SRR027236	SRR027237	SRR027238	SRR027239	SRR027240	SRR027241
SRR027242	SRR027243	SRR027244	SRR027245	SRR027246	SRR027247	SRR027248	SRR027249	SRR027250
SRR027251	SRR027252	SRR027253	SRR027254	SRR027255	SRR027256	SRR027257	SRR027258	SRR027259
SRR027260	SRR027261	SRR027262	SRR027263	SRR027264	SRR027265	SRR027266	SRR027267	SRR027268
SRR027269	SRR027270	SRR027271	SRR027272	SRR027273	SRR027274	SRR027275	SRR027276	SRR027277
SRR027278	SRR027279	SRR027280	SRR027281	SRR027282	SRR027283	SRR027284	SRR027285	SRR027286
SRR027287	SRR027288	SRR027289	SRR027290	SRR027291	SRR027292	SRR027293	SRR027294	SRR027295
SRR027296	SRR027297	SRR027298	SRR027299	SRR027300	SRR027301	SRR027302	SRR027303	SRR027304

SRR027305	SRR027306	SRR027307	SRR027308	SRR027309	SRR027310	SRR027311	SRR027312	SRR027313
SRR027314	SRR027315	SRR027316	SRR027317	SRR027318	SRR027319	SRR027320	SRR027321	SRR027322
SRR027323	SRR027324	SRR027325	SRR027326	SRR027327	SRR027328	SRR027329	SRR027330	SRR027331
SRR027332	SRR027333	SRR027334	SRR027335	SRR027336	SRR027337	SRR027338	SRR027339	SRR027340
SRR027341	SRR027342	SRR027343	SRR027344	SRR027345	SRR027346	SRR027347	SRR027348	SRR027349
SRR027350	SRR027351	SRR027352	SRR027353	SRR027354	SRR027355	SRR027356	SRR027357	SRR027358
SRR027359	SRR027360	SRR027361	SRR027362	SRR027363	SRR027364	SRR027365	SRR027366	SRR027367
SRR027368	SRR027369	SRR027370	SRR027371	SRR027372	SRR027373	SRR027374	SRR027375	SRR027376
SRR027377	SRR027378	SRR027379	SRR027380	SRR027381	SRR027382	SRR027383	SRR027384	SRR027385
SRR027386	SRR027387	SRR027388	SRR027389	SRR027390	SRR027391	SRR027392	SRR027393	SRR027394
SRR027395	SRR027396	SRR027397	SRR027398	SRR027399	SRR027400	SRR027401	SRR027402	SRR027403
SRR027404	SRR027405	SRR027406	SRR027407	SRR027408	SRR027409	SRR027410	SRR027411	SRR027412
SRR027413	SRR027414	SRR027415	SRR027416	SRR027417	SRR027418	SRR027419	SRR027420	SRR027421
SRR027422	SRR027423	SRR027424	SRR027425	SRR027426	SRR027427	SRR027428	SRR027429	SRR027430
SRR027431	SRR027432	SRR027433	SRR027434	SRR027435	SRR027436	SRR027437	SRR027438	SRR027439
SRR027440	SRR027441	SRR027442	SRR027443	SRR027444	SRR027445	SRR027446	SRR027447	SRR027448
SRR027449	SRR027450	SRR027451	SRR027452	SRR027453	SRR027454	SRR027455	SRR027456	SRR027457
SRR027458	SRR027459	SRR027460	SRR027461	SRR027462	SRR027463	SRR027464	SRR027465	SRR027466
SRR027467	SRR027468	SRR027469	SRR027470	SRR027471	SRR027472	SRR027473	SRR027474	SRR027475
SRR027476	SRR027477	SRR027478	SRR027479	SRR027480	SRR027481	SRR027482	SRR027483	SRR027484
SRR027485	SRR027486	SRR027487	SRR027488	SRR027489	SRR027490	SRR027491	SRR027492	SRR027493
SRR027494	SRR027495	SRR027496	SRR027497	SRR027498	SRR027499	SRR027500	SRR027501	SRR027502
SRR027555	SRR027556	SRR027557	SRR027558	SRR027559	SRR027560	SRR027561	SRR027562	SRR027563
SRR027578	SRR027579	SRR027580	SRR027581	SRR027582	SRR027583	SRR027584	SRR027585	SRR027586
SRR027587	SRR027588	SRR027589	SRR027590	SRR027591	SRR027592	SRR027593	SRR027594	SRR027595
SRR027596	SRR027597	SRR027598	SRR027599	SRR027600	SRR027601	SRR027602	SRR027603	SRR027604
SRR027605	SRR027606	SRR027607	SRR027608	SRR027609	SRR027673	SRR027674	SRR027675	SRR027676
SRR027677	SRR027678	SRR027679	SRR027680	SRR027681	SRR027682	SRR027683	SRR027684	SRR027685
SRR027686	SRR027687	SRR027688	SRR027610	SRR027611	SRR027612	SRR027613	SRR027614	SRR027615
SRR027616	SRR027617	SRR027618	SRR027619	SRR027620	SRR027621	SRR027622	SRR027623	SRR027624
SRR027625	SRR027626	SRR027627	SRR027628	SRR027629	SRR027630	SRR027631	SRR027632	SRR027633
SRR027634	SRR027635	SRR027636	SRR027637	SRR027638	SRR027639	SRR027640	SRR027641	SRR027642
SRR027643	SRR027644	SRR027645	SRR027646	SRR027647	SRR027648	SRR027649	SRR027650	SRR027651
SRR027652	SRR027653	SRR027654	SRR027655	SRR027656	SRR027657	SRR027658	SRR027659	SRR027660
SRR027661	SRR027662	SRR027663	SRR027664	SRR027665	SRR027666	SRR027667	SRR027668	SRR027669
SRR027670	SRR027671	SRR027672	SRR027952	SRR027967	SRR027968	SRR027969	SRR027970	SRR027971
SRR027972	SRR027973	SRR027974	SRR027975	SRR027976	SRR027977	SRR027978	SRR027979	SRR027980
SRR027981	SRR027982	SRR027983	SRR027984	SRR027985	SRR027986	SRR027987	SRR027988	SRR027989
SRR027990	SRR027991	SRR027992	SRR027993	SRR027994	SRR027995	SRR027996	SRR027997	SRR027998
SRR028001	SRR028002	SRR028003	SRR028004	SRR028005	SRR028006	SRR028007	SRR028008	SRR028009
SRR028010	SRR028011	SRR028012	SRR028013	SRR028014	SRR028015	SRR028016	SRR028017	SRR028018
SRR028019	SRR028020	SRR028021	SRR028022	SRR028023	SRR028024	SRR028025	SRR028026	SRR028027
SRR028028	SRR028029	SRR028030	SRR028031	SRR028032	SRR028033	SRR028034	SRR028035	SRR028036
SRR028037	SRR028038	SRR028039	SRR028040	SRR028041	SRR028042	SRR028043	SRR028044	SRR028668
SRR028669	SRR028670	SRR028671	SRR028672	SRR028673	SRR028674	SRR028675	SRR028676	SRR028677
SRR028678	SRR028679	SRR028680	SRR028681	SRR028682	SRR028683	SRR028736	SRR028737	SRR028738
SRR028739	SRR028740	SRR028741	SRR028742	SRR028743	SRR028744	SRR028745	SRR028746	SRR028747
SRR028748	SRR028749	SRR028750	SRR028751	SRR028752	SRR028753	SRR028754	SRR028755	SRR028756
SRR029056	SRR029057	SRR029058	SRR029059	SRR029060	SRR029061	SRR029062	SRR029063	SRR029064
SRR029065	SRR029066	SRR029067	SRR029068	SRR029069	SRR029070	SRR029071	SRR029072	SRR029073
SRR029074	SRR029075	SRR029076	SRR029077	SRR029078	SRR029079	SRR029080	SRR029081	SRR029082
SRR029083	SRR029084	SRR029085	SRR029086	SRR029087	SRR029088	SRR029089	SRR029090	SRR029091
SRR029092	SRR029093	SRR029094	SRR029095	SRR029096	SRR029097	SRR029098	SRR029099	SRR029100
SRR029101	SRR029102	SRR031972	SRR031973	SRR031974	SRR031975	SRR031976	SRR031977	SRR031978
SRR031979	SRR031980	SRR031981	SRR031982	SRR031983	SRR031984	SRR031985	SRR031986	SRR031987
SRR031988	SRR034455	SRR034456	SRR034457	SRR034458	SRR036384	SRR036385	SRR036386	SRR036380
SRR036381	SRR036382	SRR036383	SRR036387	SRR036388	SRR036389	SRR037008	SRR038651	SRR039271
SRR039272	SRR039273	SRR039274	SRR039275	SRR039276	SRR039277	SRR039278	SRR039279	SRR039280
SRR039281	SRR039282	SRR039283	SRR039284	SRR039285	SRR039286	SRR039287	SRR039288	SRR039289
SRR039290	SRR039291	SRR039292	SRR039293	SRR039294	SRR039295	SRR039296	SRR039297	SRR039298
SRR039299	SRR039300	SRR039301	SRR039302	SRR039303	SRR039304	SRR039305	SRR039306	SRR039307

SRR039308	SRR039309	SRR039310	SRR039311	SRR039312	SRR039910	SRR064542	SRR066139	SRR070081
SRR070082	SRR070083	SRR070084	SRR091237	SRR091238	SRR094454	SRR391540	SRR094455	SRR391542
SRR094456	SRR391543	SRR095679	SRR118420	SRR189064	SRR192554	SRR275326	SRR306100	SRR306101
SRR304671	SRR304672	SRR304673	SRR304674	SRR304680	SRR324677	SRR333051	SRR333052	SRR333053
SRR333054	SRR333055	SRR333056	SRR333057	SRR333058	SRR333059	SRR333060	SRR333061	SRR333062
SRR333063	SRR333064	SRR333065	SRR333066					
**marine sediment metagenome**								
DRR001556	DRR001557	SRR001322	SRR001323	SRR001324	SRR001325	SRR001326	SRR023396	SRR038652
SRR043581	SRR043582	SRR070884	SRR070885	SRR070886	SRR070887	SRR070888	SRR070889	SRR070890
SRR070891	SRR070892	SRR070893	SRR070894	SRR070895	SRR070896	SRR070897	SRR070898	SRR070899
SRR070900	SRR070901	SRR070902	SRR070903	SRR070904	SRR070905	SRR070906	SRR070907	SRR071118
SRR071119	SRR071120	SRR071121	SRR071123	SRR071124	SRR071125	SRR071126	SRR099552	SRR099553
SRR099554	SRR099555	SRR302362	SRR363457	SRR363458	SRR363459	SRR363460	SRR363461	SRR363462
SRR363463	SRR363464	SRR363465	SRR363466	SRR363497	SRR363498	SRR363499	SRR363500	SRR364710
SRR364674	SRR364709	SRR364702	SRR396763	SRR396765	SRR396764	SRR396767	SRR396769	SRR396768
SRR396770	SRR396771	SRR396772	SRR396773	SRR396774	SRR396775	SRR396776	SRR396777	
**microbial mat metagenome**								
DRR000019	DRR000020	DRR000021	DRR000358					
**mine drainage metagenome**								
SRR290507	SRR290508	SRR290509	SRR290510					
**mosquito metagenome**								
SRR192322	SRR192323	SRR192324	SRR192325	SRR192326	SRR192327	SRR192556	SRR192662	SRR192664
SRR192777	SRR192878	SRR192879	SRR192880	SRR192883	SRR192884	SRR192886	SRR192929	SRR192930
SRR192931	SRR192932	SRR192933	SRR192934	SRR192935	SRR192936			
**mouse gut metagenome**								
ERR016007	ERR016008	ERR047491	ERR047492	SRR031071	SRR031072	SRR031073	SRR063774	SRR063853
SRR064443	SRR058830	SRR058831	SRR058832	SRR058833	SRR058834	SRR058835	SRR058836	SRR058837
SRR058838	SRR058839							
**oil production facility metagenome**								
SRR203252	SRR354054	SRR360720	SRR360838	SRR360839	SRR360840	SRR360841	SRR360842	SRR360843
SRR360844	SRR360845	SRR360846	SRR360847	SRR360848	SRR360849	SRR360850	SRR360851	
**phyllosphere metagenome**								
ERR042936	ERR058011	ERR058012	ERR058013	ERR058014	ERR058015	ERR058016	ERR058017	ERR058018
ERR058019	ERR058020	ERR058021	ERR058022	ERR058023	ERR058024	ERR058025	ERR058026	ERR058027
ERR058028	ERR058029	ERR058030	ERR058031	ERR058032	ERR058033	ERR058034	ERR058035	ERR058036
ERR058040	ERR058041	ERR058043	ERR058044	ERR058045	ERR058046	SRR023845	SRR023846	
**rhizosphere metagenome**								
ERR034493	ERR034494	ERR066891	ERR066892	ERR066893	ERR066894	ERR066895	ERR066896	ERR066897
ERR066898	ERR066899	ERR066900	SRR358565					
**root metagenome**								
SRR385744	SRR385745							
**saltern metagenome**								
SRR001046	SRR001053	SRR001054	SRR001055	SRR001056	SRR001057	SRR001058	SRR001059	SRR001060
SRR023631	SRR027043	SRR062267	SRR316684	SRR328982	SRR328983			
**sediment metagenome**								
SRR074105	SRR074106	SRR088761	SRR088762	SRR088763	SRR088764	SRR088765	SRR088768	SRR088766
SRR088767	SRR089801	SRR089809	SRR089810	SRR089811	SRR089812	SRR089813	SRR089814	SRR089815
SRR097494	SRR099064	SRR326940	SRR326941	SRR326939	SRR340011	SRR340036	SRR340037	SRR340038
SRR340039	SRR340040	SRR340187	SRR341930	SRR364873	SRR364874	SRR364713	SRR364714	SRR364715
SRR364716	SRR364717	SRR364718	SRR364875	SRR364876	SRR364877	SRR364879	SRR364880	SRR364711
SRR364712	SRR364969	SRR364970	SRR364971	SRR364972	SRR364973	SRR364974	SRR364975	SRR364976
SRR364983	SRR364984	SRR364881	SRR364882	SRR364977	SRR364978	SRR364893	SRR364892	SRR364891
SRR364890	SRR364889	SRR364888	SRR364887	SRR364886	SRR364885	SRR364884	SRR364883	SRR364695
SRR364696	SRR364703	SRR364704	SRR364682	SRR364683	SRR364684	SRR364685	SRR364686	SRR364687
SRR364688	SRR364689	SRR364690	SRR364691	SRR364692	SRR364693	SRR364694	SRR385607	
**soil metagenome**								
DRR001521	DRR001522	DRR001523	DRR001524	DRR001525	DRR001526	DRR001527	DRR001528	DRR001529
DRR001530	DRR001531	DRR001532	DRR001533	DRR001534	DRR001535	DRR001536	DRR001537	DRR001538
DRR001539	DRR001540	DRR001541	DRR001542	DRR001543	DRR001544	DRR001545	DRR001546	DRR001547
DRR001548	DRR001549	DRR001550	DRR001983	DRR002212	ERR003029	ERR003030	ERR011388	ERR011389

ERR011409	ERR011393	ERR011407	ERR011397	ERR011411	ERR011387	ERR011402	ERR011404	ERR011413
ERR011396	ERR011415	ERR011401	ERR011386	ERR011391	ERR011394	ERR011403	ERR011408	ERR011410
ERR011399	ERR011414	ERR011416	ERR011417	ERR011412	ERR011406	ERR011390	ERR011405	ERR011400
ERR011395	ERR011398	ERR011392	ERR023719	ERR023720	ERR023721	ERR023722	ERR023724	ERR023723
ERR024306	ERR024296	ERR024300	ERR024315	ERR024316	ERR024304	ERR024310	ERR024295	ERR024305
ERR024309	ERR024299	ERR024312	ERR024308	ERR024294	ERR024313	ERR024297	ERR024301	ERR024302
ERR024303	ERR024298	ERR024317	ERR024314	ERR024307	ERR024311	ERR037046	ERR037053	ERR037062
ERR037075	ERR037045	ERR037050	ERR037052	ERR037061	ERR037047	ERR037051	ERR037055	ERR037059
ERR037056	ERR037060	ERR037067	ERR037071	ERR037058	ERR037065	ERR037070	ERR037057	ERR037064
ERR037069	ERR037073	ERR037048	ERR037049	ERR037063	ERR037072	ERR037054	ERR037066	ERR037068
ERR039004	ERR039005	ERR039006	ERR039007	ERR039008	ERR039009	ERR039010	ERR039011	ERR039012
ERR039013	ERR039014	ERR039015	ERR039515	ERR039514	ERR039523	ERR039516	ERR039519	ERR039517
ERR039522	ERR039521	ERR039520	ERR039518	ERR046706	ERR046707	ERR046708	ERR046709	ERR054050
ERR054053	ERR054052	ERR054049	ERR054048	ERR054051	ERR054054	ERR055326	ERR055327	ERR055328
SRR015218	SRR023819	SRR023820	SRR023821	SRR036794	SRR054583	SRR059807	SRR061001	SRR059808
SRR061002	SRR059809	SRR061003	SRR059810	SRR061004	SRR060996	SRR061005	SRR060997	SRR061006
SRR060998	SRR061007	SRR060999	SRR061008	SRR061000	SRR061009	SRR064356	SRR064357	SRR064358
SRR064371	SRR064370	SRR064372	SRR064373	SRR064374	SRR064375	SRR064376	SRR064377	SRR064378
SRR064379	SRR064380	SRR064381	SRR064382	SRR064383	SRR064384	SRR066669	SRR066670	SRR066671
SRR066672	SRR066673	SRR066674	SRR171663	SRR171664	SRR171305	SRR171307	SRR171684	SRR090314
SRR090399	SRR091626	SRR094400	SRR094717	SRR095659	SRR124473	SRR125367	SRR125368	SRR125369
SRR125370	SRR125371	SRR125372	SRR125373	SRR125374	SRR125528	SRR125529	SRR125550	SRR125607
SRR125608	SRR125609	SRR125610	SRR125611	SRR125612	SRR125613	SRR125614	SRR125615	SRR125616
SRR125617	SRR125618	SRR125619	SRR125623	SRR139146	SRR139149	SRR139150	SRR139151	SRR205889
SRR260097	SRR360834	SRR360810	SRR360881	SRR360932				
**stromatolite metagenome**								
SRR001043	SRR001044	SRR001045	SRR001061	SRR001062	SRR001063	SRR329490		
**termite gut metagenome**								
SRR388113								
**termite metagenome**								
SRR332541	SRR332542	SRR332543	SRR332544	SRR359013	SRR359014	SRR359015	SRR359016	
**wasp metagenome**								
SRR332546	SRR332547	SRR332548	SRR359009	SRR359010	SRR359011	SRR359012		
**wastewater metagenome**								
SRR001308	SRR017891	SRR017892	SRR017893	SRR017894	SRR017895	SRR017896	SRR017897	SRR017898
SRR022927	SRR040812	SRR042235	SRR042236	SRR042394	SRR042395	SRR042396	SRR042397	SRR042398
SRR042399	SRR057574	SRR057575	SRR057576	SRR057577	SRR057578	SRR069841	SRR069842	SRR069843
SRR069844	SRR069845	SRR069846	SRR069847	SRR069848	SRR069849	SRR069850	SRR069851	SRR069852
SRR069853	SRR069854	SRR069855	SRR069856					
